# Supplementary material for: A universal strategy towards high–energy aqueous multivalent–ion batteries
Source: Nat Commun. 2021 May 17;12:2857. doi: 10.1038/s41467-021-23209-6 (PMC8128864; doi:10.1038/s41467-021-23209-6)
Supplement: Supplementary file 1 — Supplementary Information [file 41467_2021_23209_MOESM1_ESM.docx]

**Supplementary Information**

**A universal strategy towards high–energy aqueous multivalent–ion batteries**

Tang *et al.*

**Supplementary Figures**


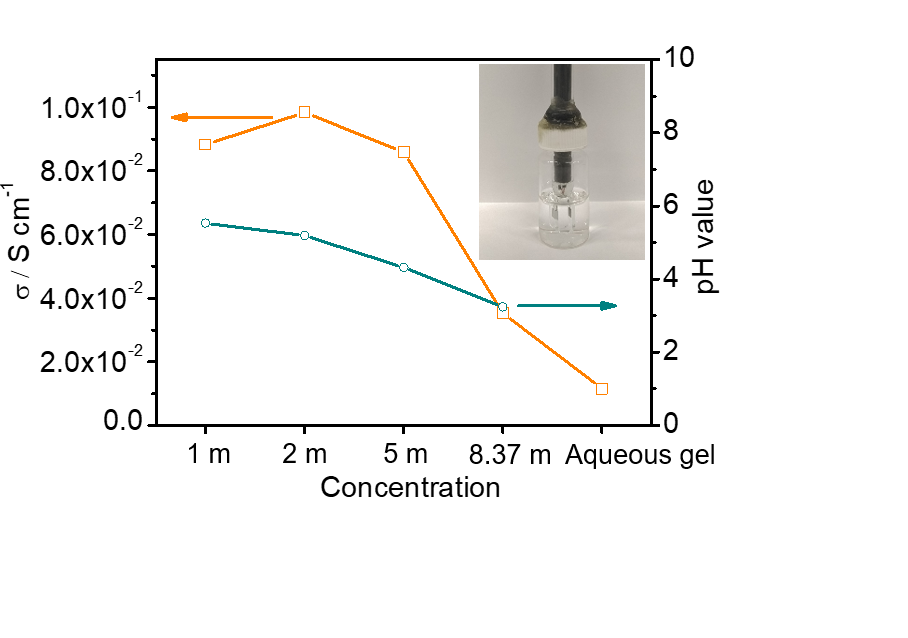


**Supplementary Figure 1**. The electrochemical conductivities and pH values of the electrolyte samples. Aqueous gel in this work is in quasi–solid–state, thus its pH value cannot be measured by the conductivity meter due to its high viscosity and stickiness. The inset shows the schematic illustration of ionic conductivity testing process via conductivity meter.


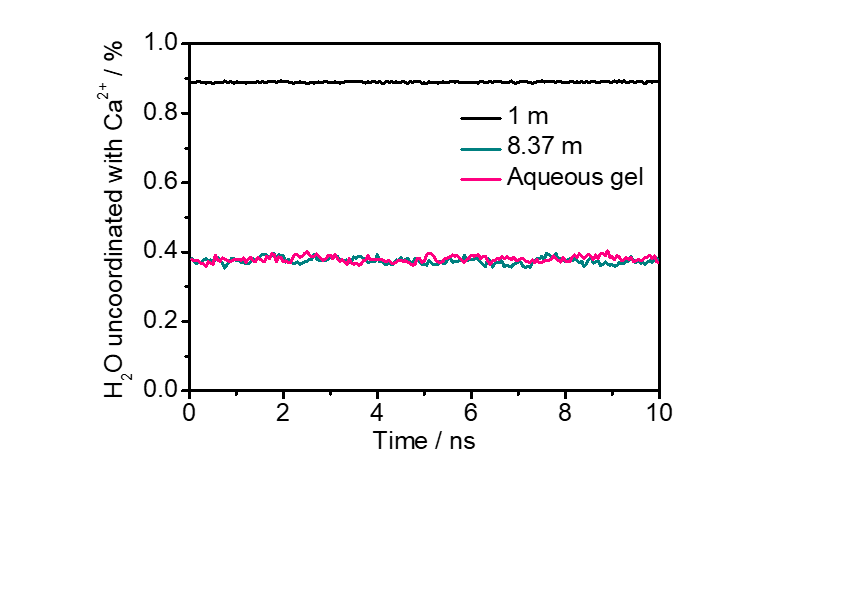


**Supplementary Figure 2**. The percentage of water molecular uncoordinated with Ca^2+^ in the three electrolyte samples based on MD simulation.


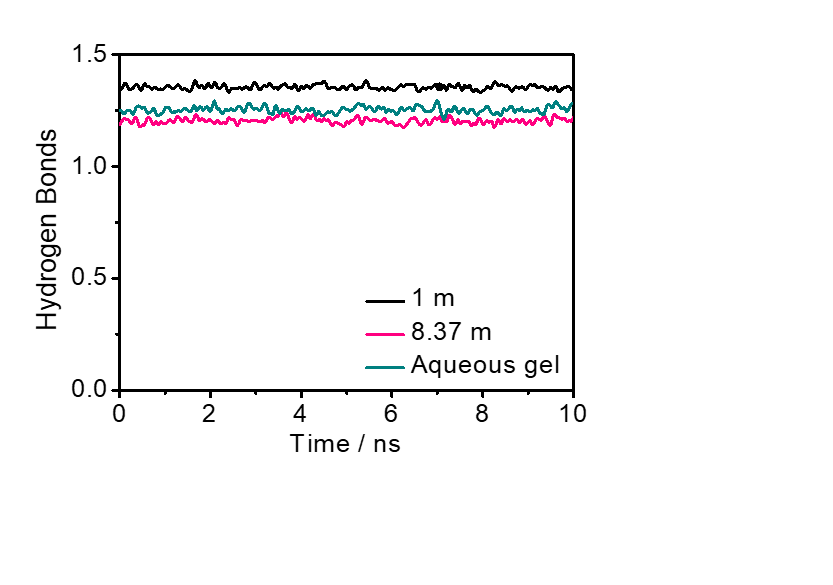


**Supplementary Figure 3**. The hydrogen bonds of the three electrolyte samples based on MD simulation.


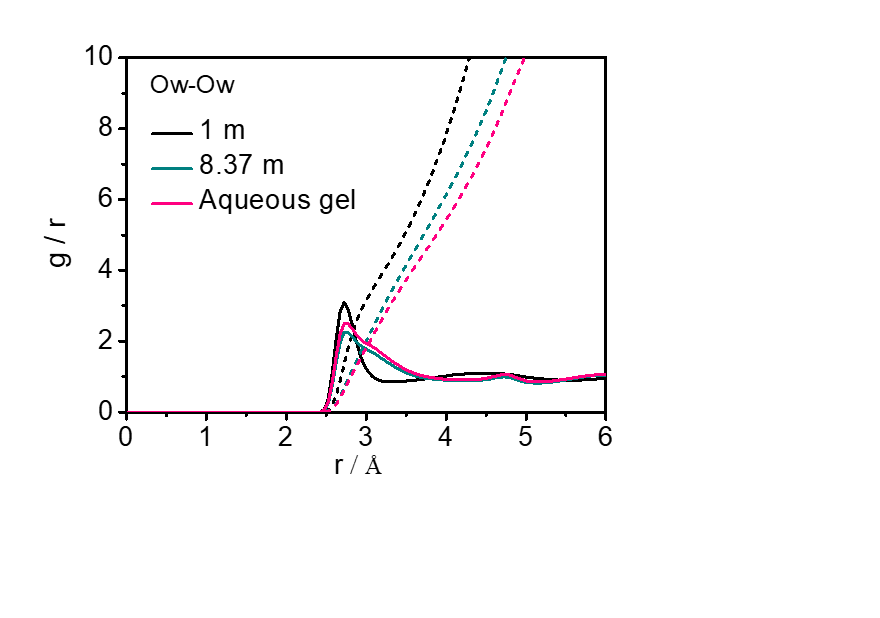


**Supplementary Figure 4.** The radial distribution functions (solid line) and its number integral (dash line) of the distance between two oxygen atoms in water molecules in different electrolytes (See discussion in Supplementary Note 1).


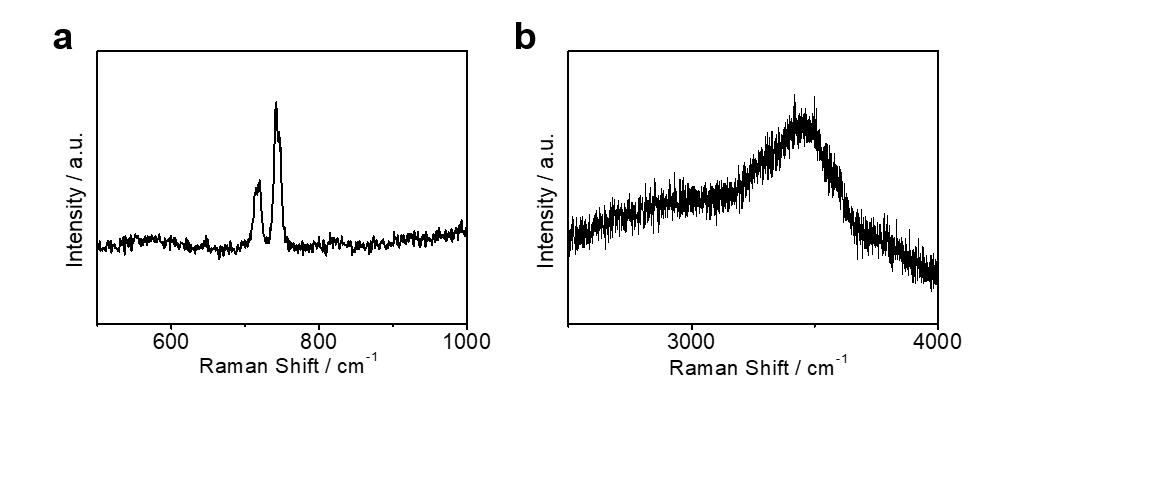


**Supplementary Figure 5**. The Raman spectra of the crystalline Ca(NO_3_)_2_·4H_2_O.


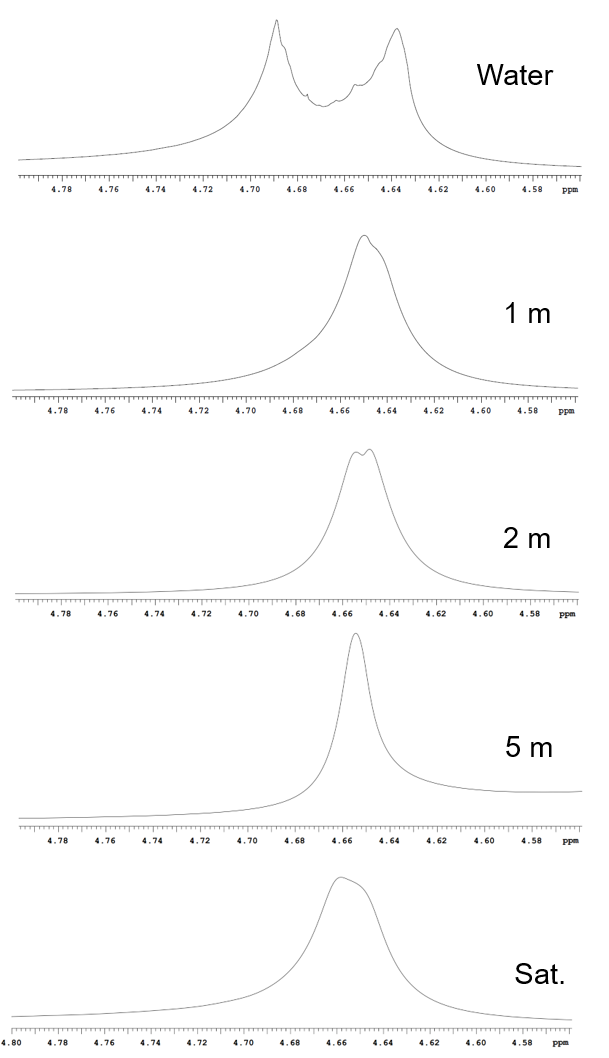


**Supplementary Figure 6**. ^1^H NMR of the pure water, 1 m, 2 m, 5 m, Sat. Ca(NO_3_)_2_ aqueous solutions.


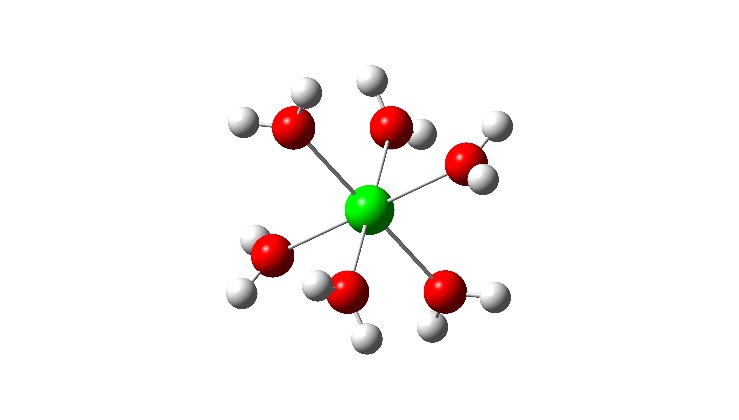


**Supplementary Figure 7.** DFT calculation of the binding energy between one water molecule and Ca^2+^ (See discussion in Supplementary Note 2).


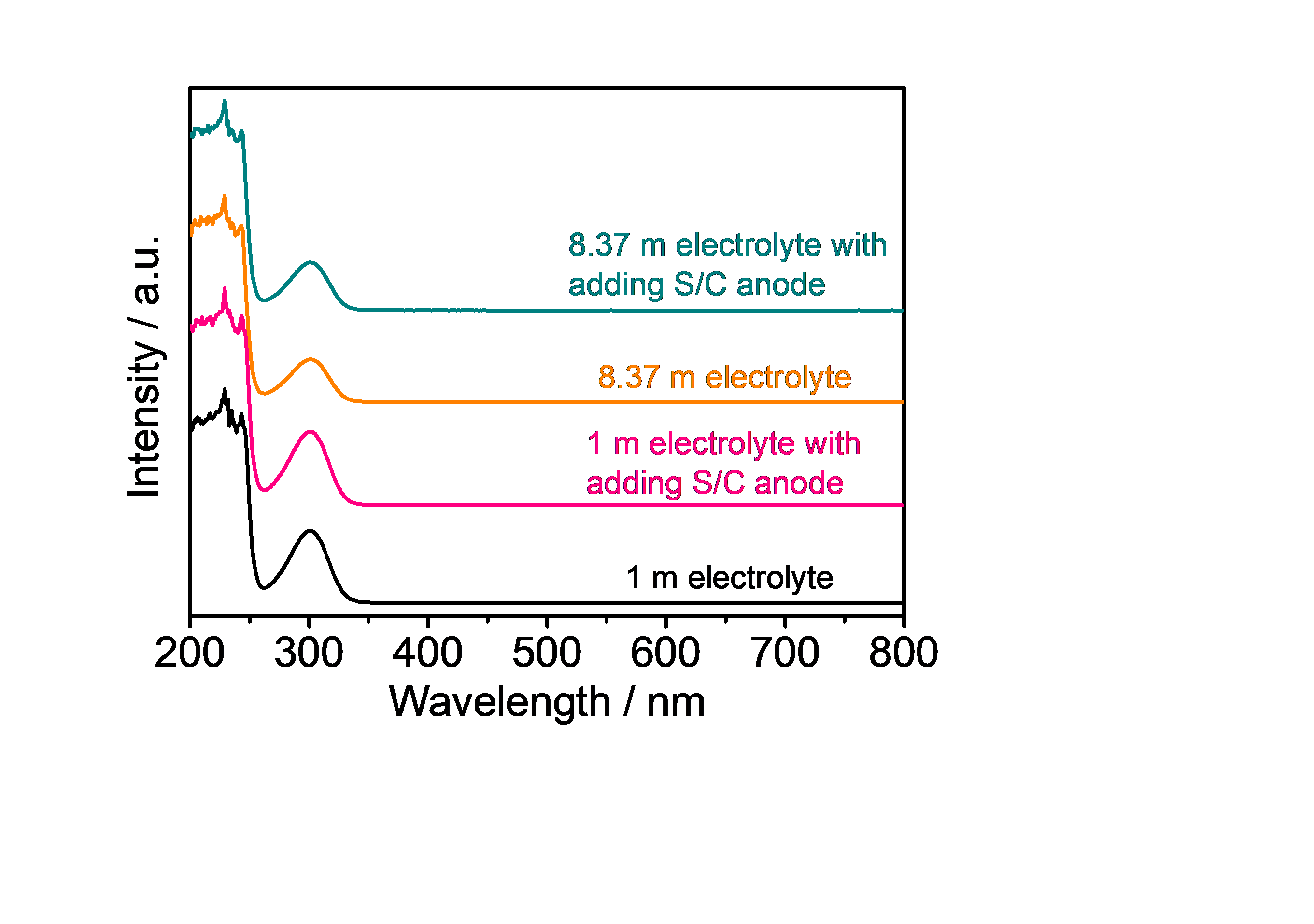


**Supplementary Figure 8**. The UV–vis spectra of the bare electrolytes and electrolytes after immersing with S/C anode (See discussion in Supplementary Note 3).


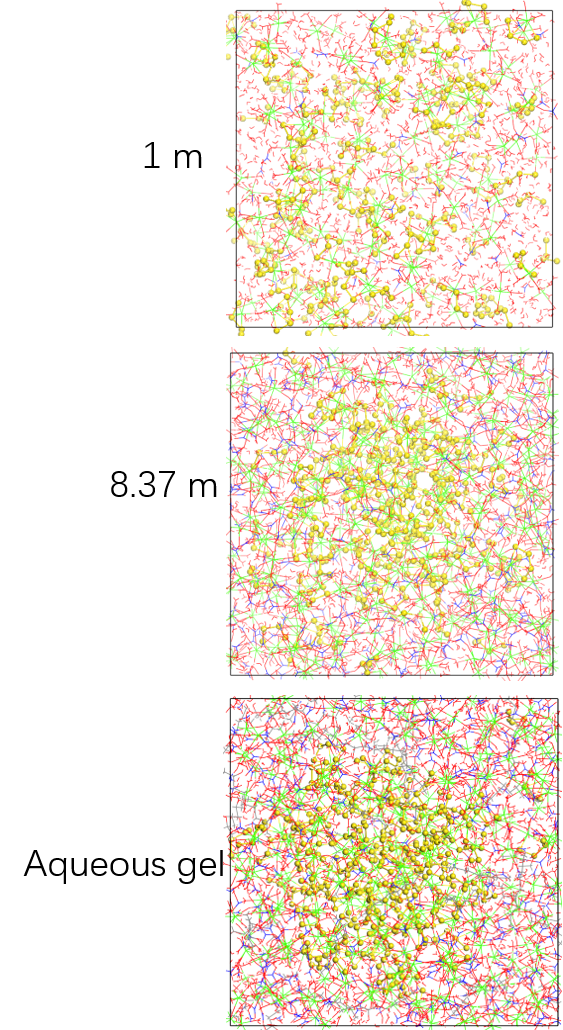


**Supplementary Figure 9**. Snapshots of CaS_4_ diffusion in 1 m, Saturated Ca(NO_3_)_2_ solution, and gel electrolyte based on MD simulation in 5 ns.


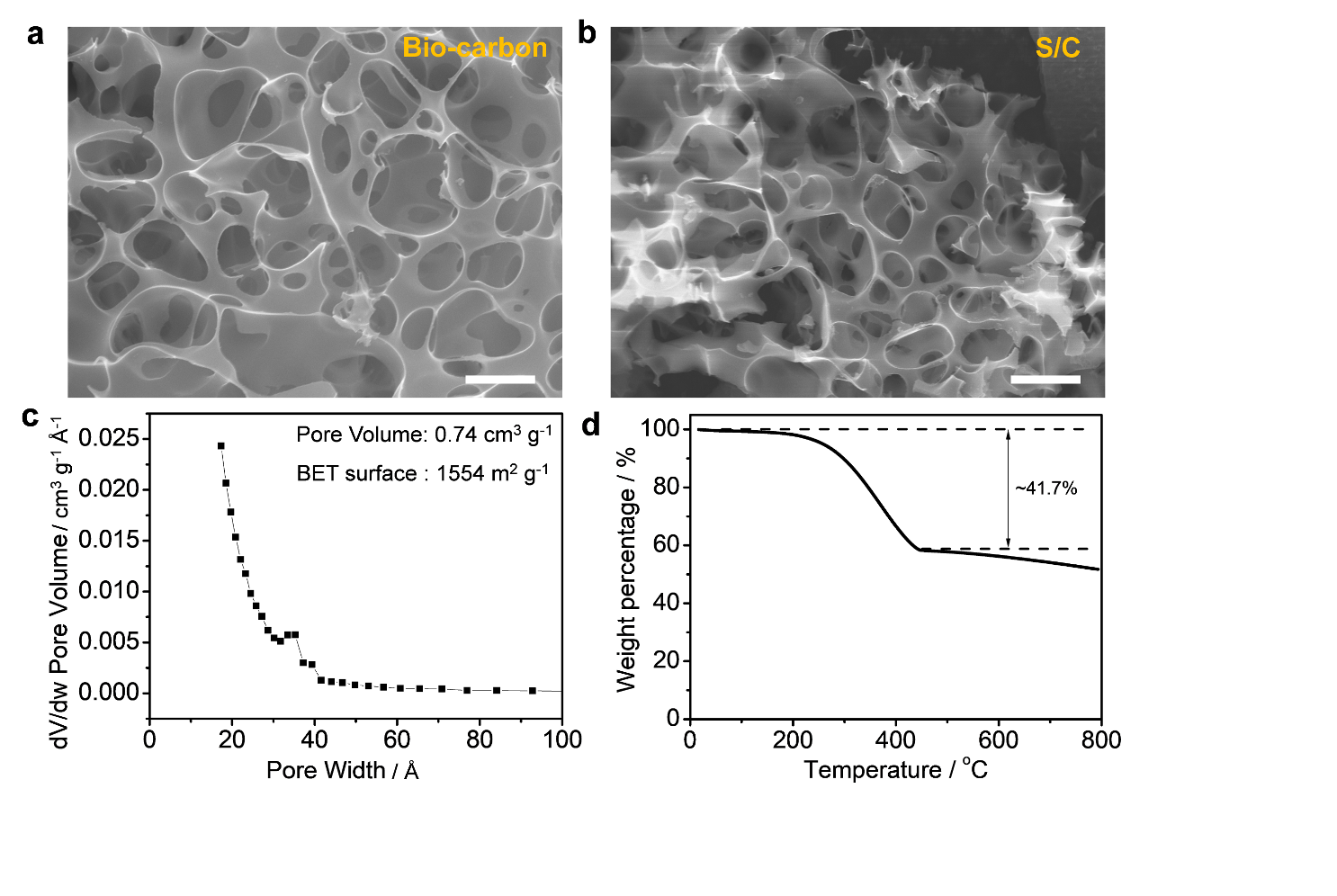


**Supplementary Figure 10**. Scanning electron microscope (SEM) images of **a** biomass–derived porous carbon host and **b** S/C composite. Scale bars are 2 μm in Supplementary Fig. 10a–b. **c** Pore distribution of the porous carbon host. **d** The thermogravimetry analysis (TGA) curve of the S/C composite under N_2_ flow. The sulfur loading in the S/C was set as ≈40 wt%, which is close to the maximum loading value (48 wt%) that guarantees the CaS as reaction product fully confined in the carbon host (considering the density of CaS is 2.83 g cm^–3^ and the pore volume of porous carbon is 0.74 cm^3^ g^–1^ according to the BET results).


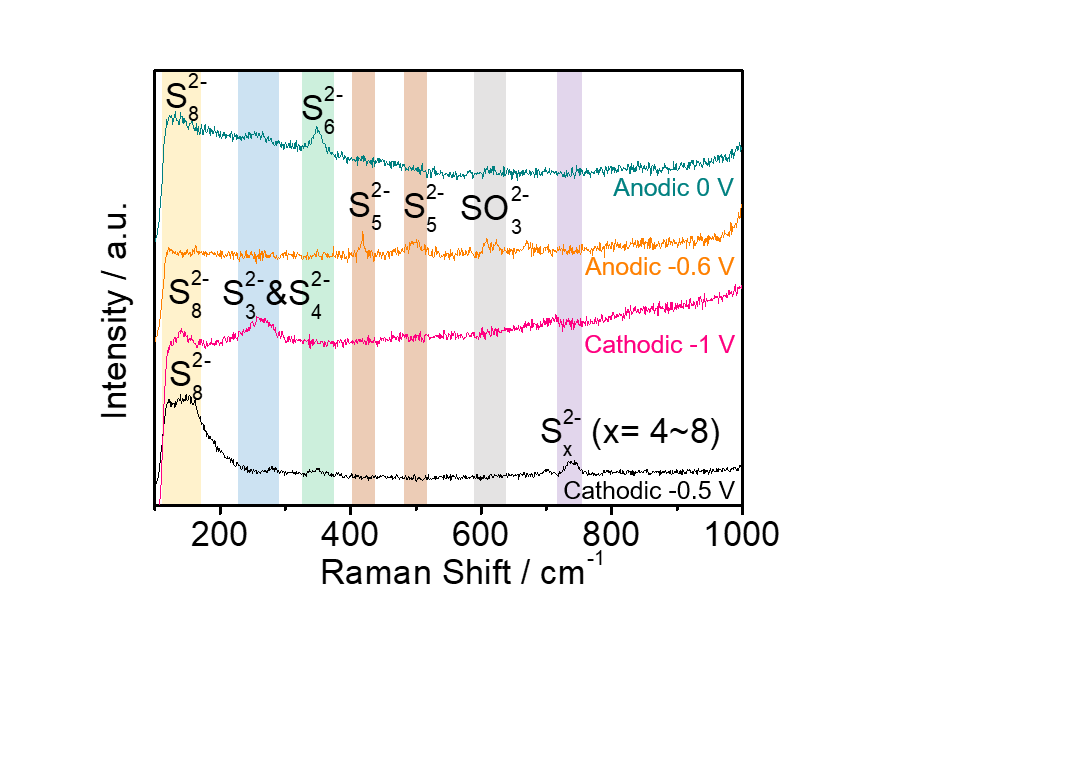


**Supplementary Figure 11**. Ex situ Raman spectra of the S/C electrodes at different discharge/charge states corresponding to Fig. 3c.


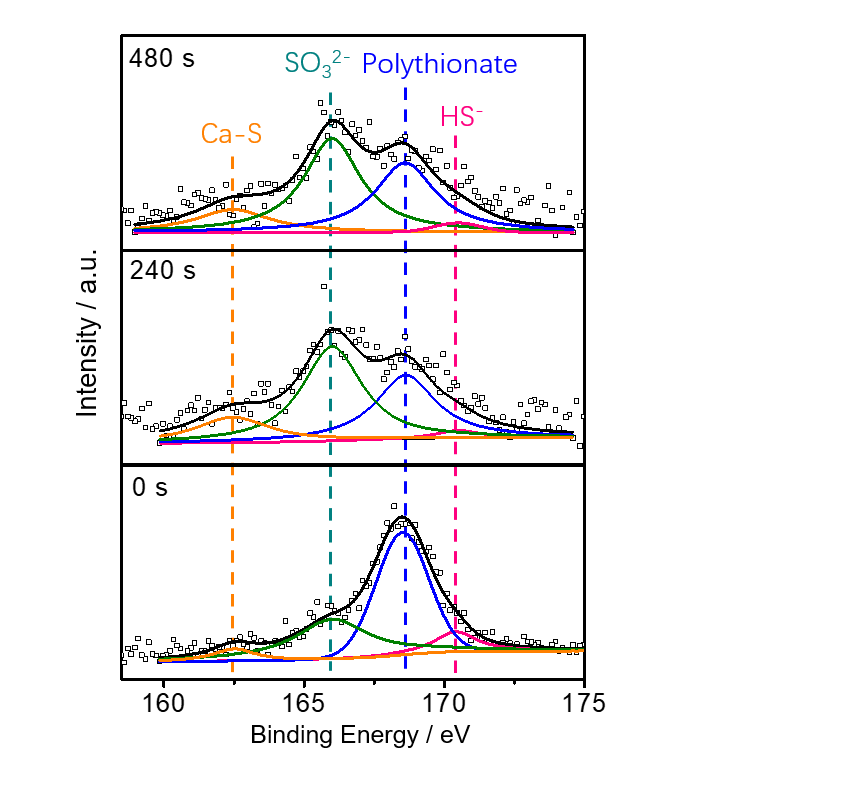


**Supplementary Figure 12**. The in–depth S 2*p* XPS of the S/C anode after the cathodic CV scanning process (See discussion in Supplementary Note 4).


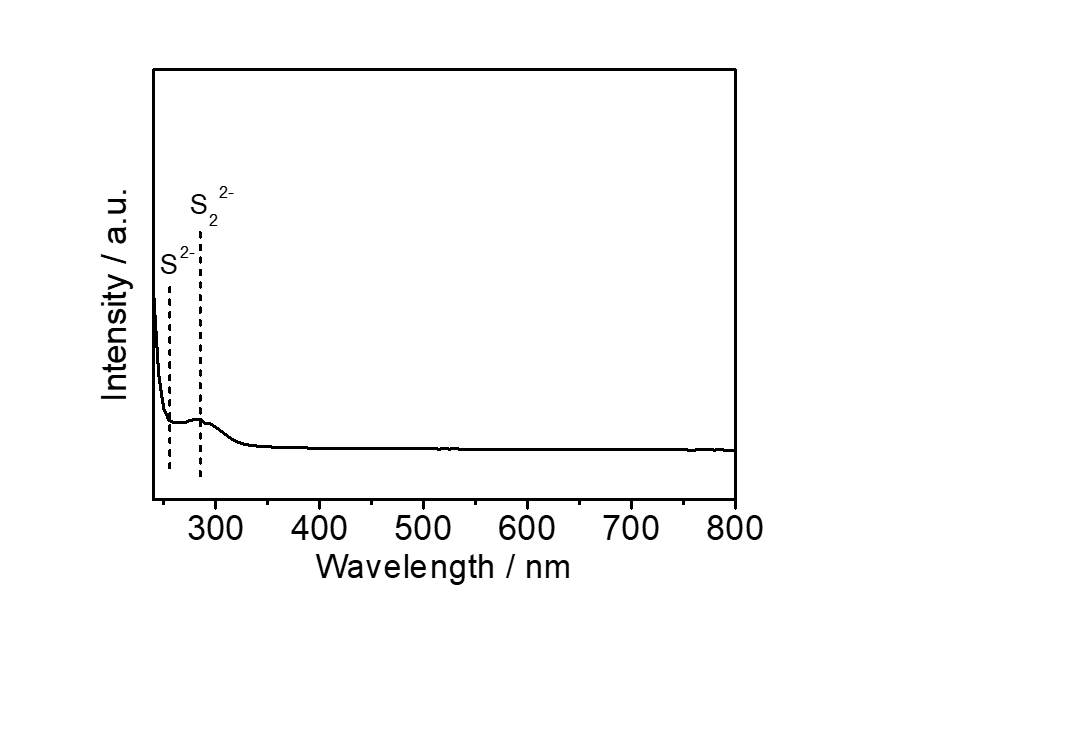


**Supplementary Figure 13**. The UV–vis spectrum of the sulfur electrode after cathodic scanning process. The as–obtained sulfur electrode was washed with 1,3–Dioxolane (DOL), and the DOL/calcium polysulfides mixture was collected for UV–vis characterization. Various Ca polysulfides (S^2−^ and S_2_^2−^ at 220~280 nm^1^) appear in the UV–Vis spectrum of DOL/Ca polysulfides mixture, which is highly consistent with the Raman and in–depth S 2*p* XPS spectra results.


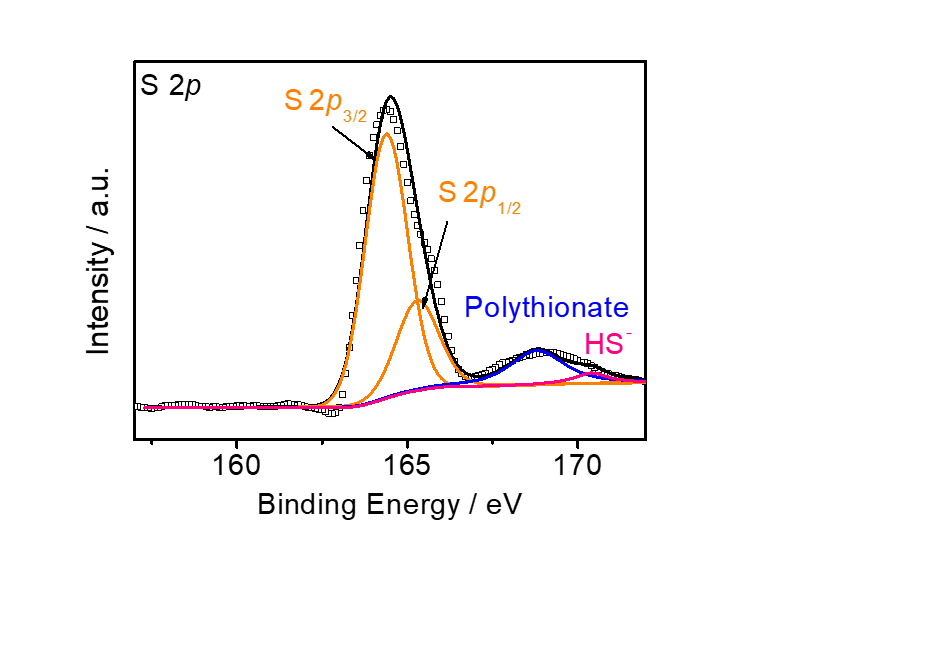


**Supplementary Figure 14**. The S 2*p* XPS of the S/C anode after one CV scanning process at a scan rate of 0.5 mV s^–1^. Two peaks at ≈164.3 and ≈165.3 eV are assigned to S 2*p*_3/2_ and S 2*p*_1/2_^2^.


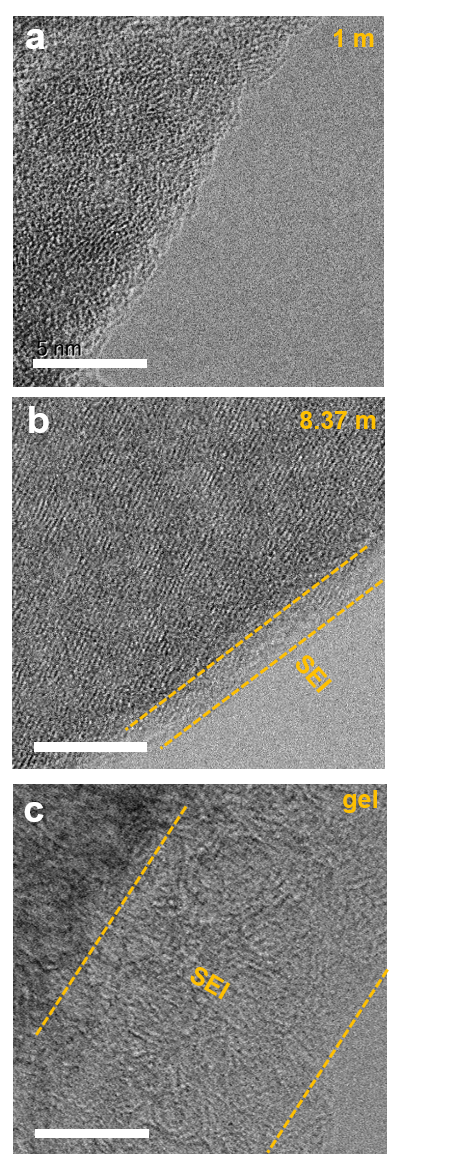


**Supplementary Figure 15**. HADDF–STEM images of the S/C electrode after a CV scanning in **a** 1 m, **b** 8.37 m Ca(NO_3_)_2_ aqueous electrolytes, and **c** aqueous gel electrolyte. The scale bars are 10 nm in Supplementary Fig. 15a–c.


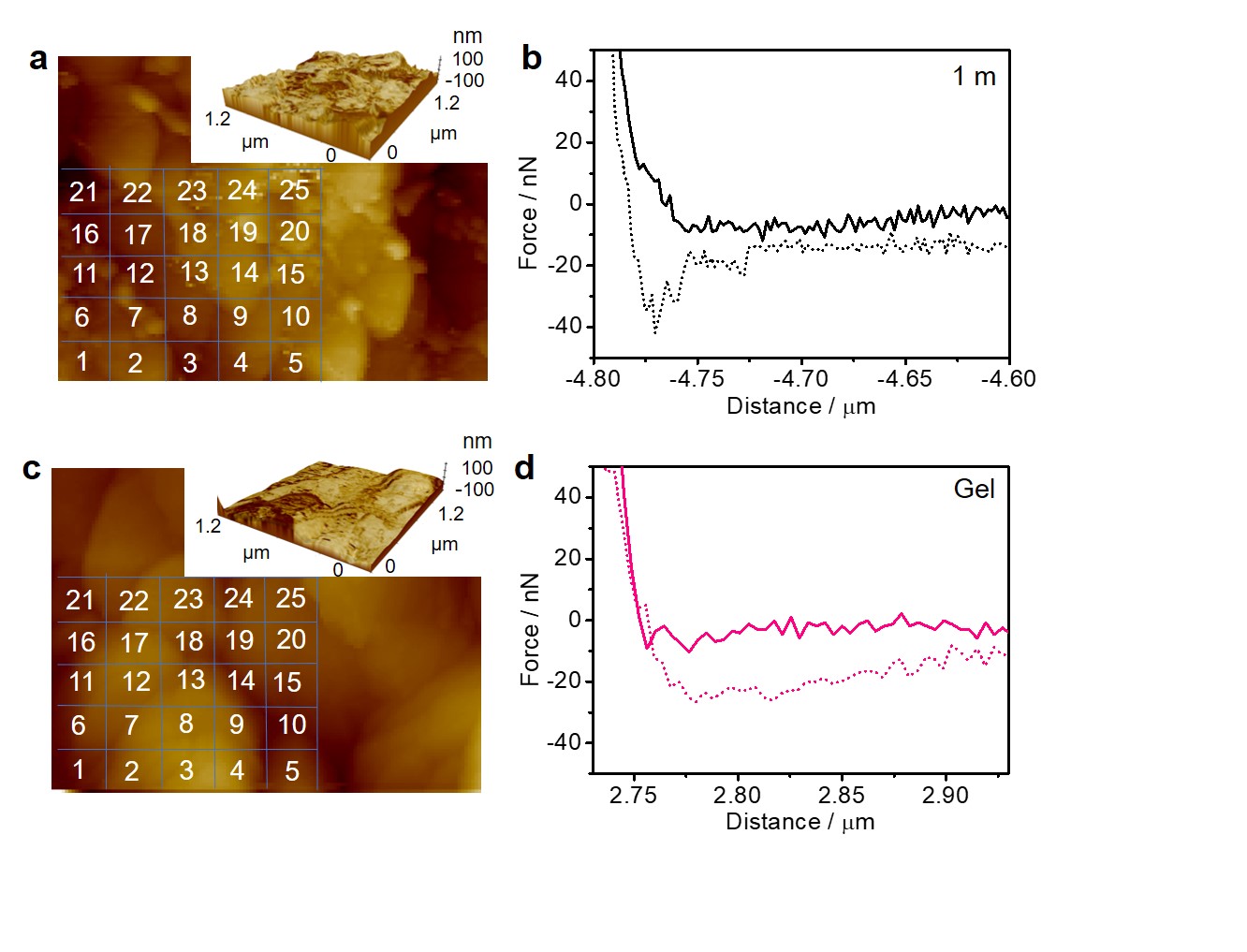


**Supplementary Figure 16**. **a** The AFM scanning images of S/C anodes tested in 1 m Ca(NO_3_)_2_ electrolyte. The corresponding three–dimensional AFM scanning images is shown in inset. **b** The corresponding force−displacement plots of area 18 in supplementary Fig. 16a. **c** The AFM scanning images of S/C anodes tested in aqueous gel electrolyte. The corresponding three–dimensional AFM scanning image is shown in inset. **d** The corresponding force−displacement plots of area 18 in supplementary Fig. 16c.


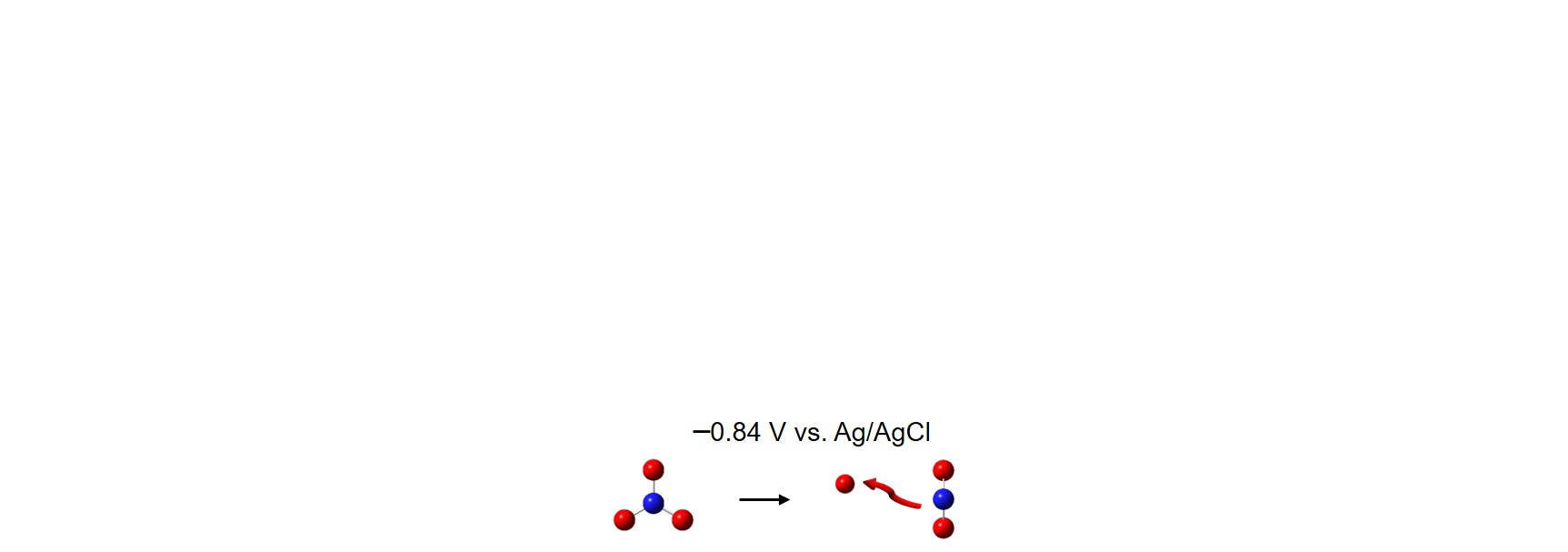


**Supplementary Figure 17.** The DFT calculations of reduction of isolated NO_3_^–^.


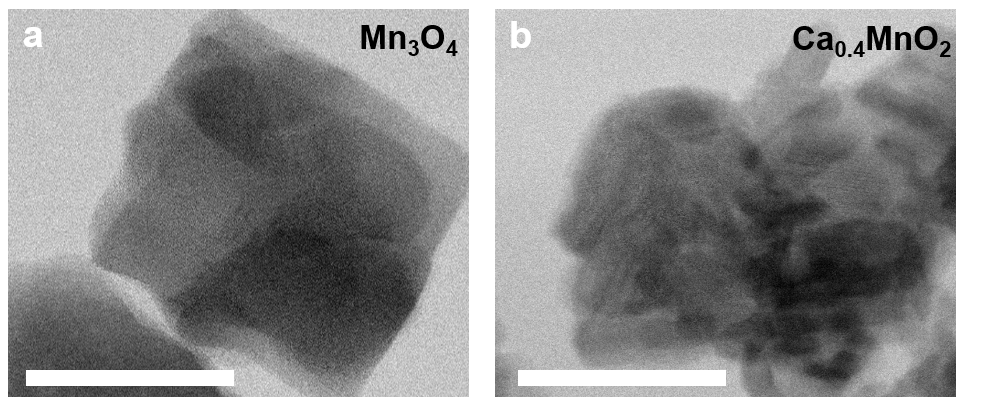


**Supplementary Figure 18.** The TEM images of the as–prepared **a** Mn_3_O_4_ precursor and **b** Ca_0.4_MnO_2_ cathode material. Scale bars: 50 nm.


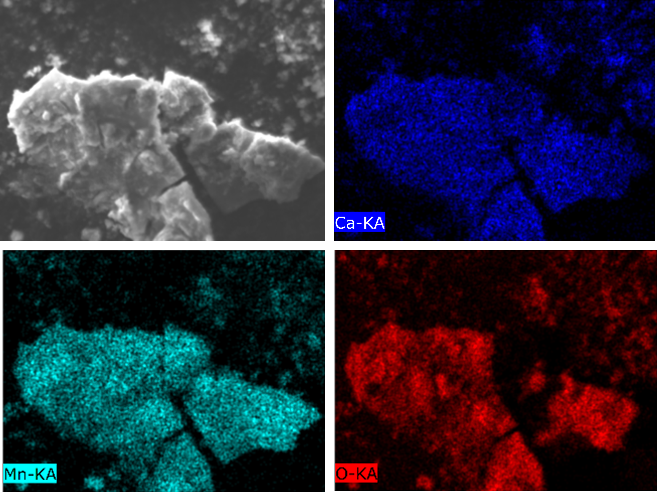


**Supplementary Figure 19.** The elemental mapping of Ca, Mn, O in the Ca_0.4_MnO_2_ cathode material.


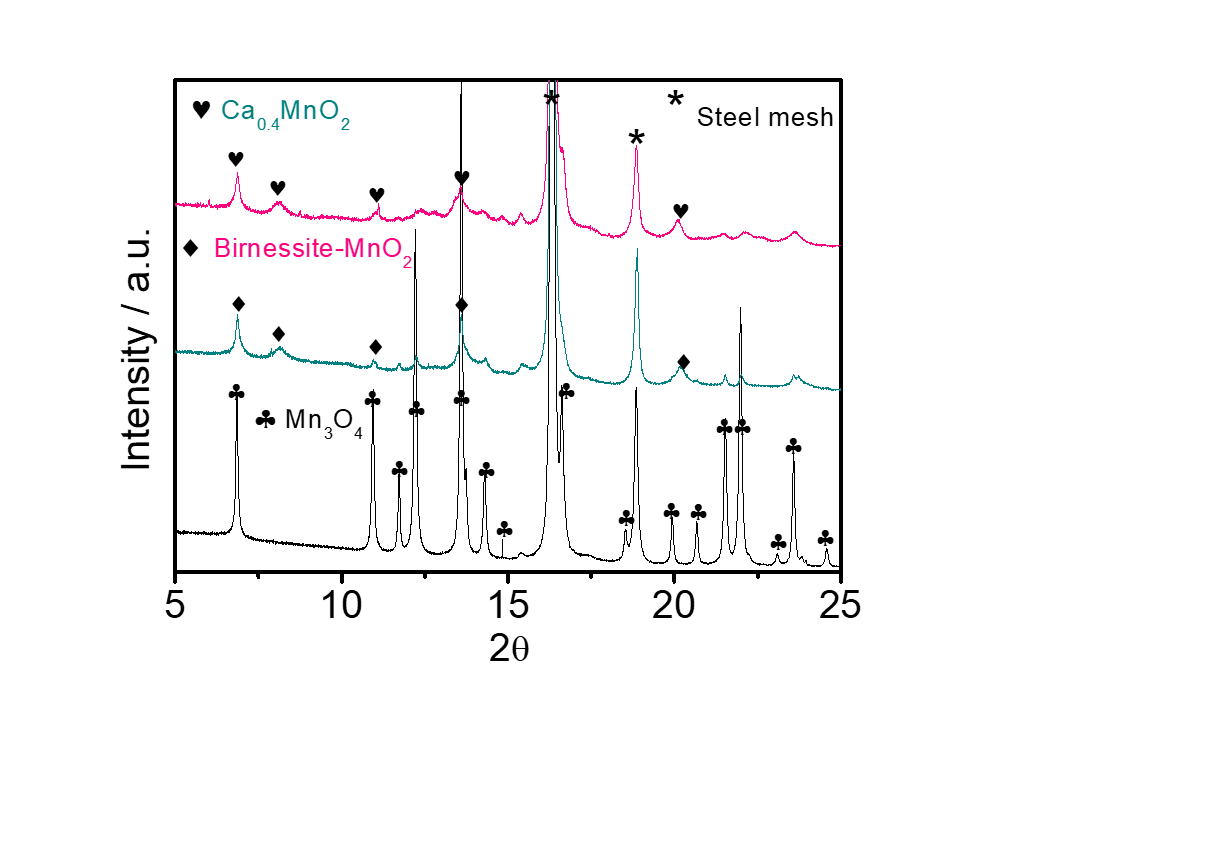


**Supplementary Figure 20.** The synchrotron X–ray powder diffraction patterns of Mn_3_O_4_, birnessite MnO_2_ and Ca_0.4_MnO_2_.


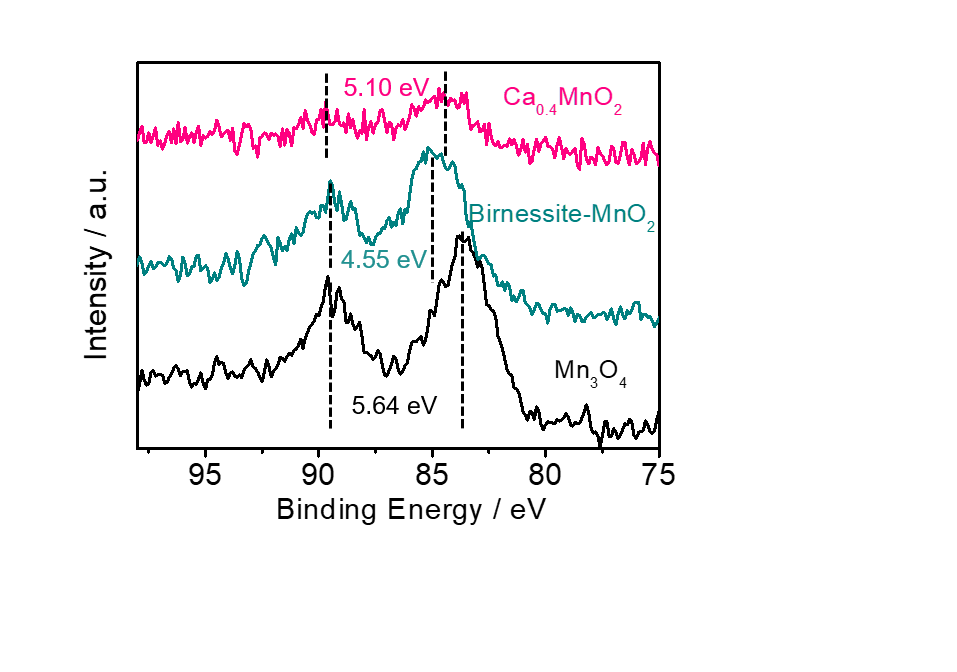


**Supplementary Figure 21.** The Mn 3*s* XPS spectra of the Mn_3_O_4_, birnessite MnO_2_ and Ca_0.4_MnO_2_.


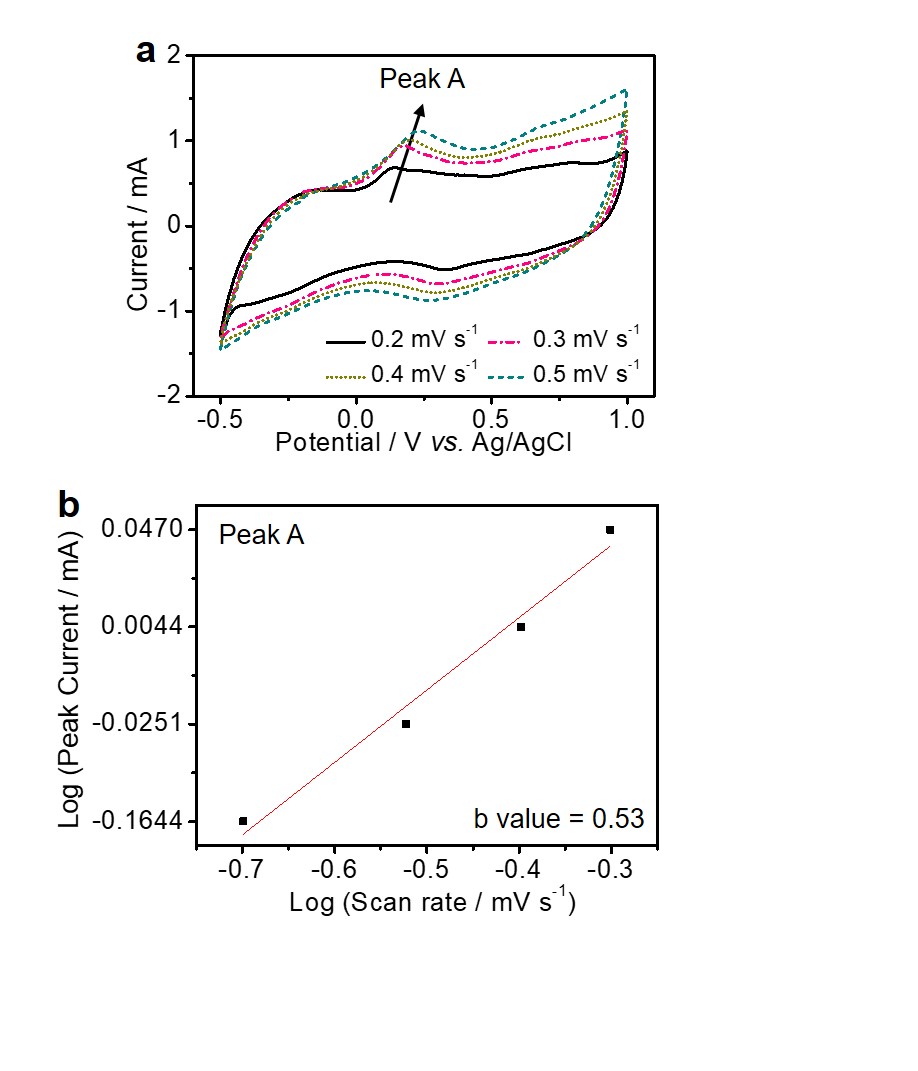


**Supplementary Figure 22.** **a** The CV curves of the Ca_0.4_MnO_2_ cathode collected at scan rates of 0.2~0.5 mV s^–1^. **b** Plot of log(peak current) as function of log(scan rate) obtained from Supplementary Figure 22a (See discussion in Supplementary Note 5).


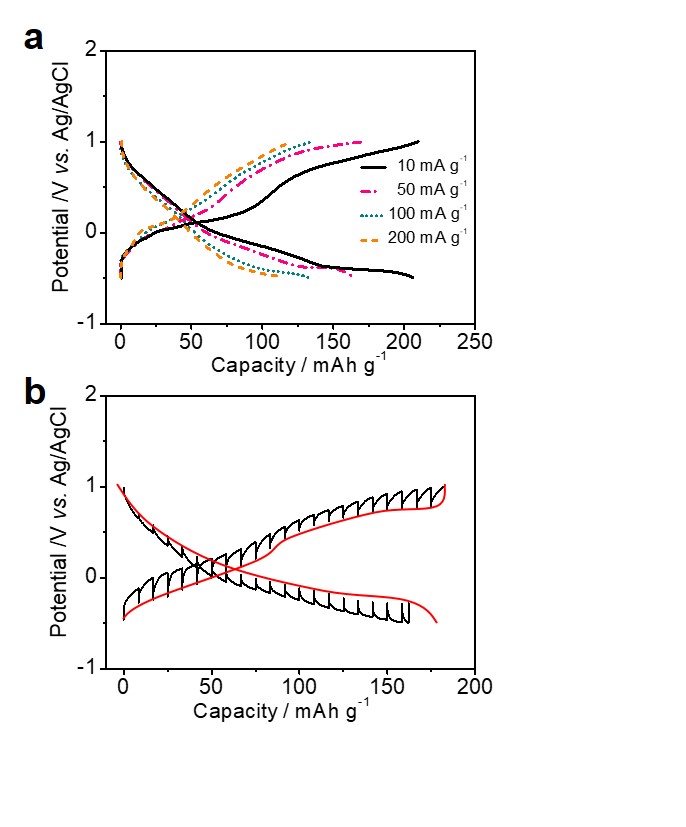


**Supplementary Figure 23.** **a** Voltage profiles of the Ca_0.4_MnO_2_ cathode in 8.37 m Ca(NO_3_)_2_ aqueous electrolyte at different specific currents. **b** The GITT curves of the Ca_0.4_MnO_2_ cathode.


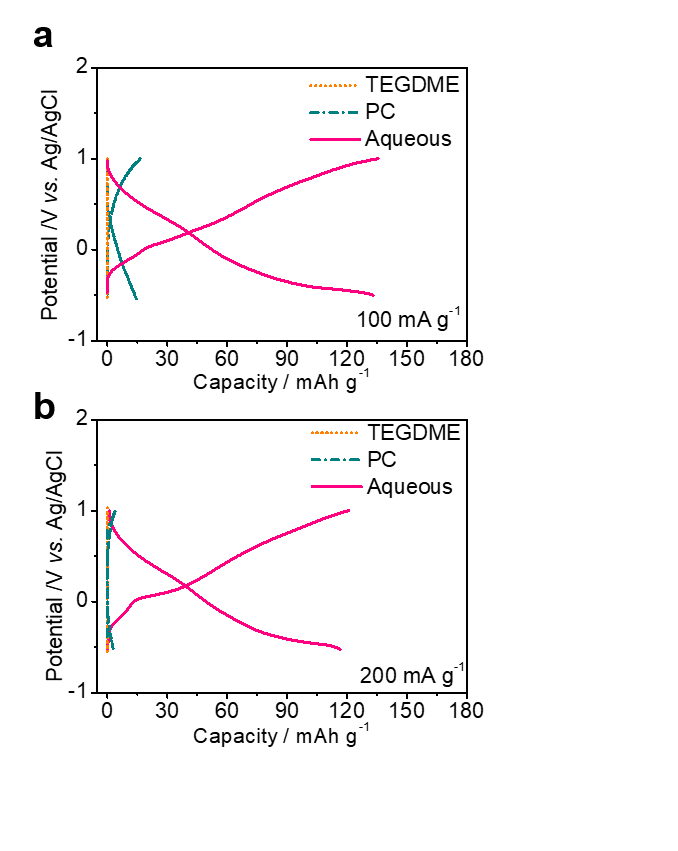


**Supplementary Figure 24.** Voltage profiles of the Ca_0.4_MnO_2_ cathode tested in 0.5 m Ca(OTf)_2_ in TEGDME, 0.5 m Ca(OTf)_2_ in PC, and 8.37 m Ca(NO_3_)_2_ aqueous electrolytes at specific current of **a** 100 mA g^–1^ and **b** 200 mA g^–1^.


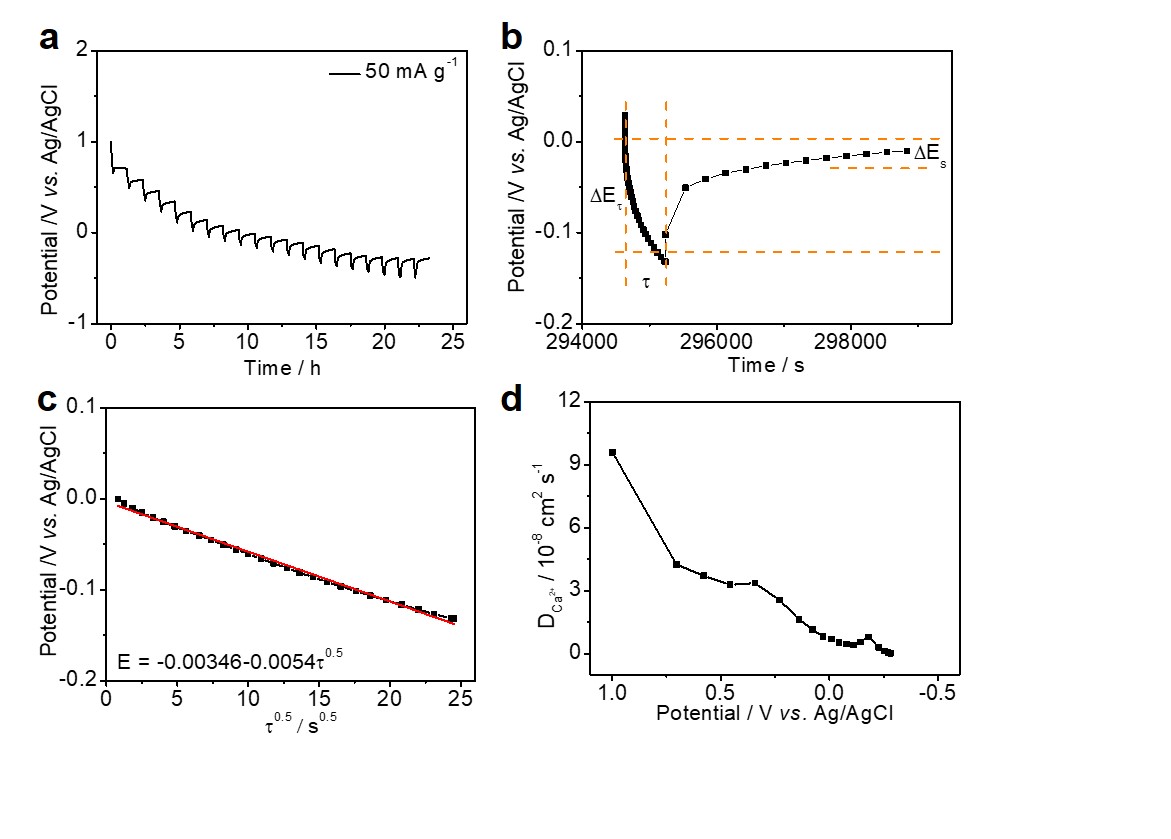


**Supplementary Figure 25.** Characterization of the Ca^2+^ diffusion co–efficiency in Ca_0.4_MnO_2_ cathode. **a–b** GITT curves of the Ca_0.4_MnO_2_ cathode. **c** Plot of potential as function of τ^0.5^ obtained from Supplementary Fig. 25b. **d** Plot of Ca^2+^ diffusion co–efficiency as function of potential (See discussion in Supplementary Note 6).


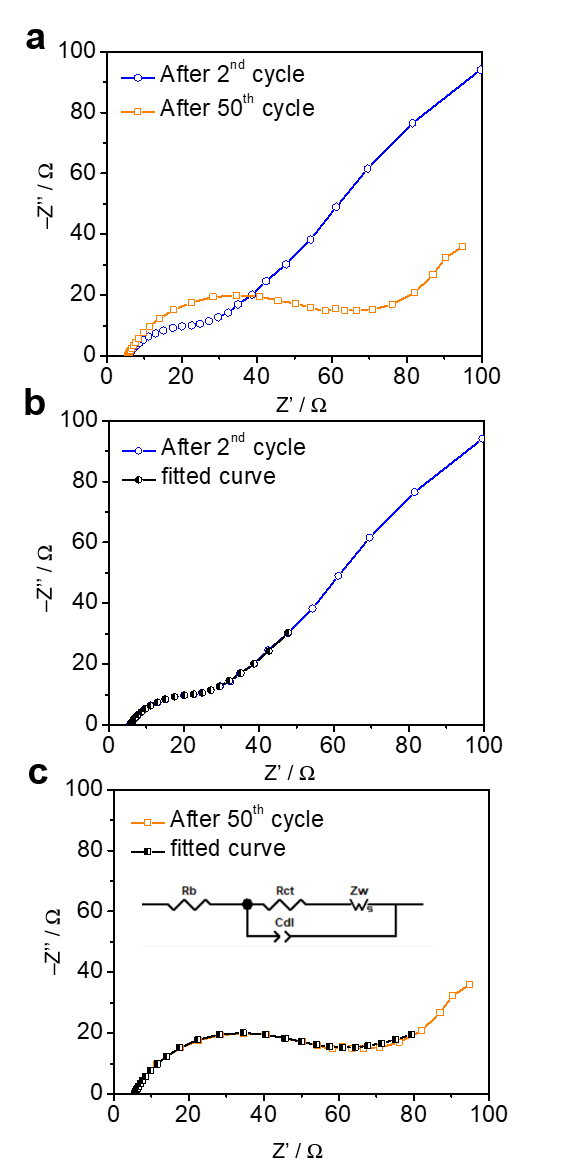


**Supplementary Figure 26.** **a** The EISs of the Ca_0.4_MnO_2_ cathode after different cycles. **b–c** The fitted EIS curves of the as–prepared Ca_0.4_MnO_2_ cathode based on an equivalent circuit shown as inset (See discussion in Supplementary Note 7).


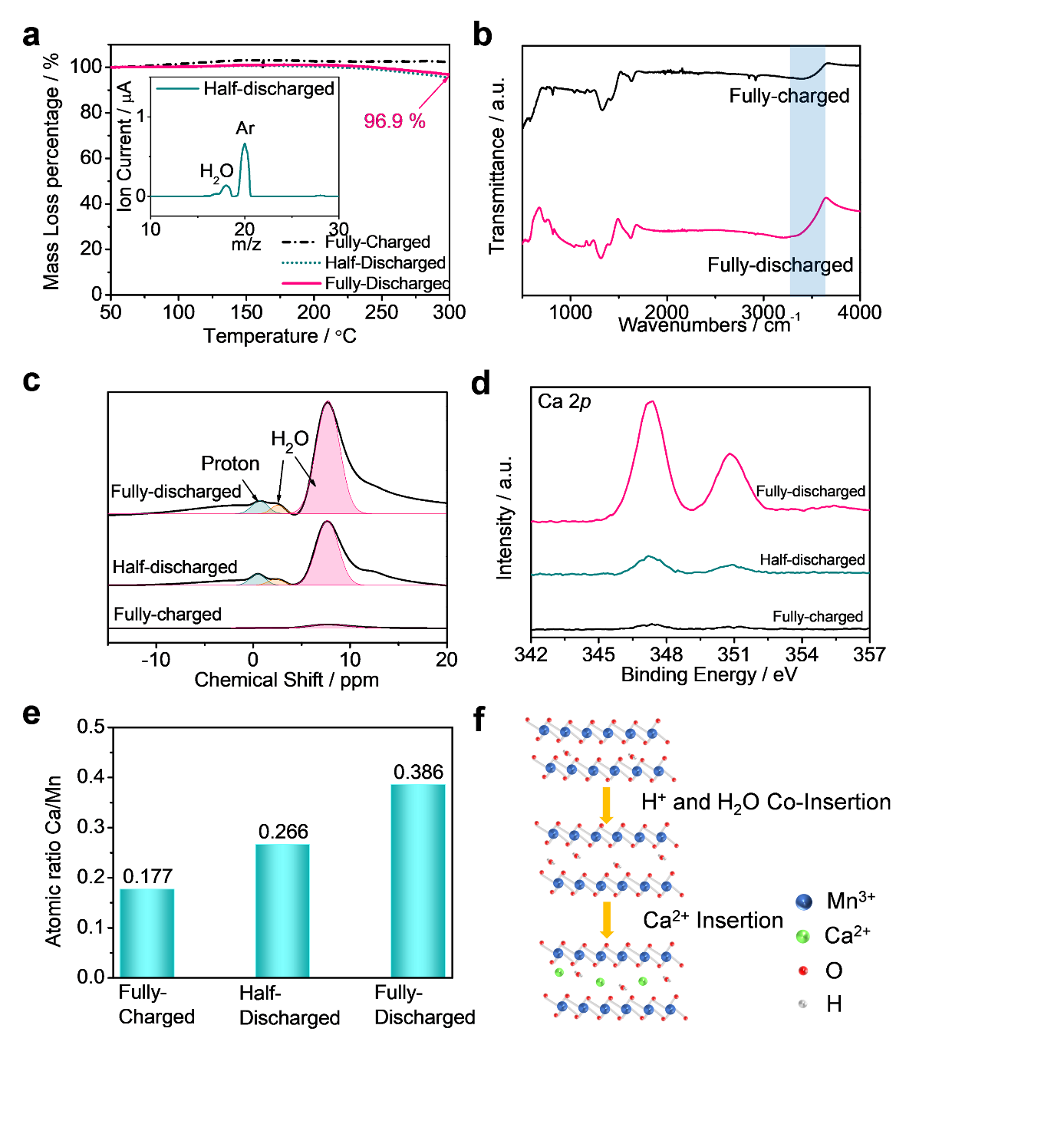


**Supplementary Figure 27.** H^+^/Ca^2+^ co–intercalation chemistry in Ca_0.4_MnO_2_ cathode in aqueous gel electrolyte. The cathode samples were pre–heated at 80 °C for one day before characterizations. **a** The TGA curves of the Ca_0.4_MnO_2_ cathode materials after discharge to different states of charge at 100 mA g^–1^ at under Ar flow. The inset is the mass spectrum of the evaporating gas flow from the cathode at the half–discharged state. **b** The FTIR spectra of the cathode materials at fully–charged and fully–discharged states. **c** ^1^H SSNMR spectra of the cathode materials at different states of charge. **d** XPS spectra of the cathode materials at different states of charge. **e** The Ca/Mn atomic ratio of the cathode materials at different states of charge according to the ICP results. **f** Schematic illustration the electrochemical mechanism of the Ca_0.4_MnO_2_ cathode (See discussion in Supplementary Note 8).


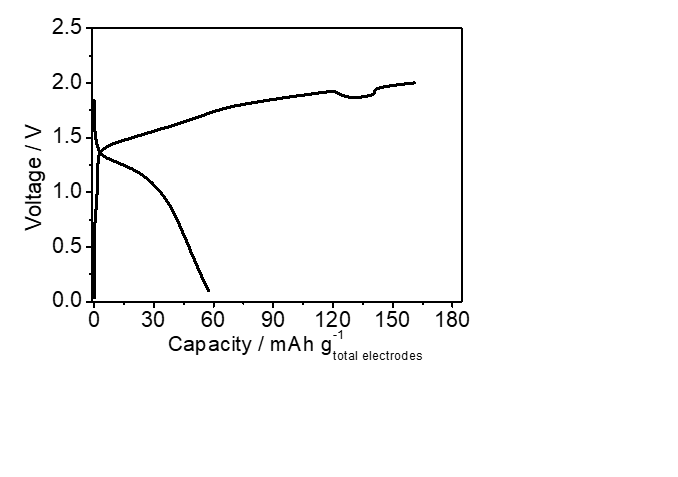


**Supplementary Figure 28.** The pre–activated charge/discharge profile of S/C||Ca_0.4_MnO_2_ full cells assembled with gel electrolyte at 0.5 C.


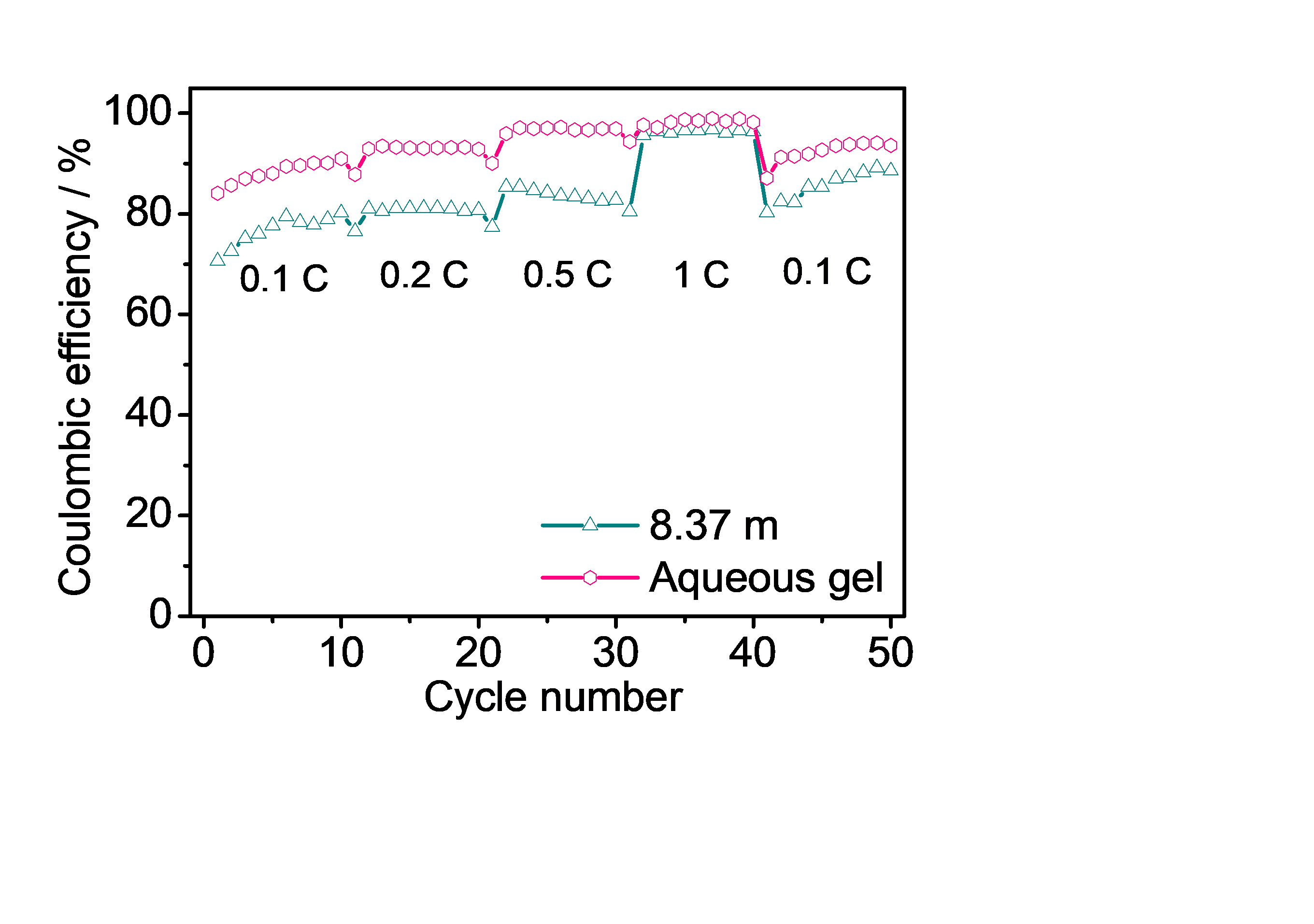


**Supplementary Figure 29.** Coulombic efficiencies of S/C||Ca_0.4_MnO_2_ full cells at different rates.


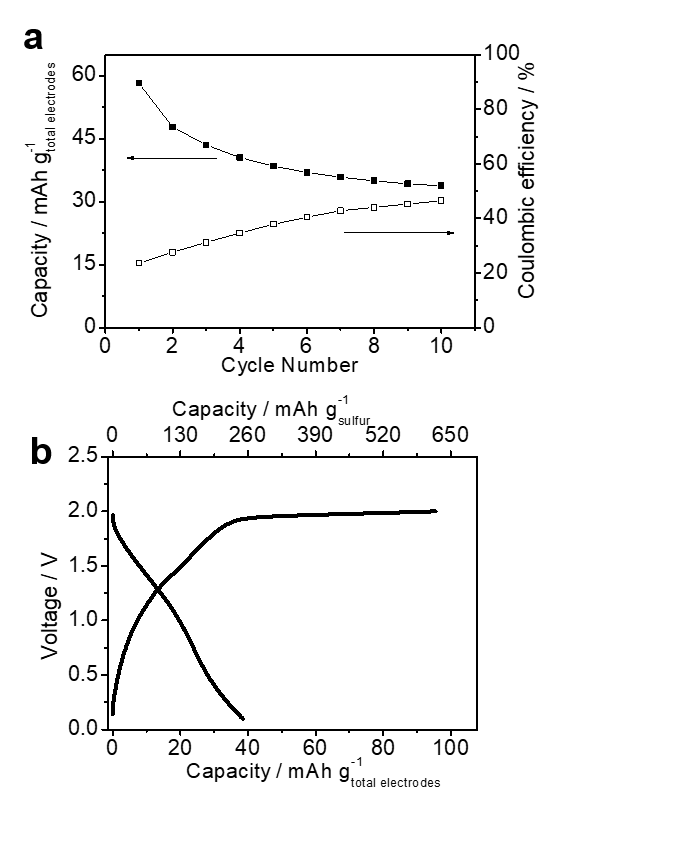


**Supplementary Figure 30.** **a** The cycling stability of the full cells at a specific current of 0.1 C. **b** The voltage profile of the S/C||Ca_0.4_MnO_2_ full cells assembled with 1 m Ca(NO_3_)_2_ electrolytes at 0.1 C.


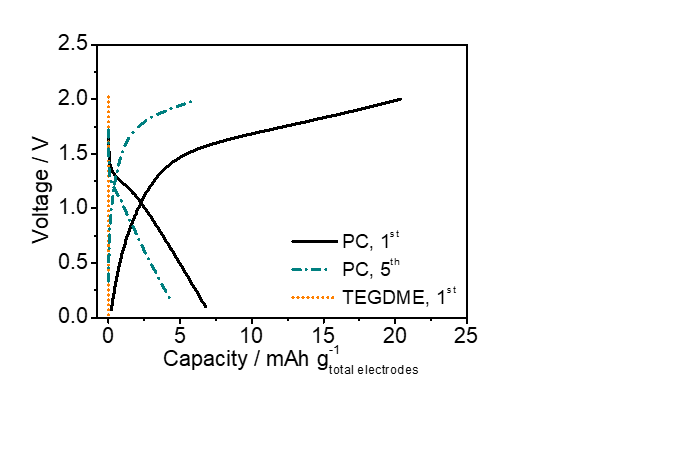


**Supplementary Figure 31.** Voltage profiles of the S/C|0.5 M Ca(OTf)_2_ in TEGDME|Ca_0.4_MnO_2_ and S/C|0.5 M Ca(OTf)_2_ in PC|Ca_0.4_MnO_2_ cells at different cycles at 0.5 C rate. The cell S/C||Ca_0.4_MnO_2_ cell cannot be cycled in the TEGDME–based electrolyte.


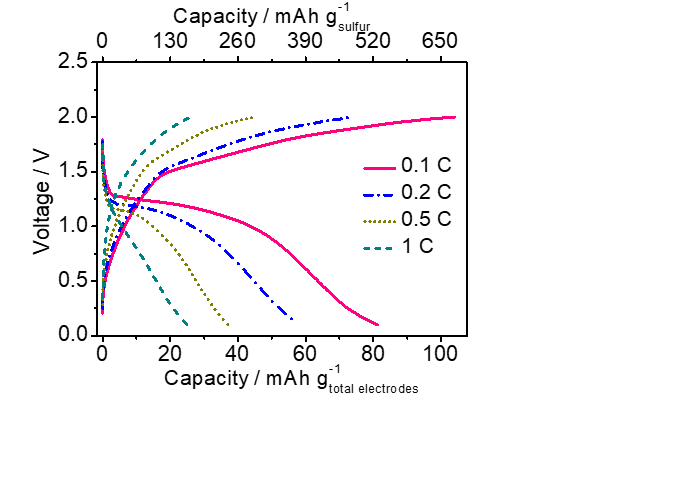


**Supplementary Figure 32**. The voltage profiles of the S/C||Ca_0.4_MnO_2_ full cells assembled with 8.37 m Ca(NO_3_)_2_ electrolytes.


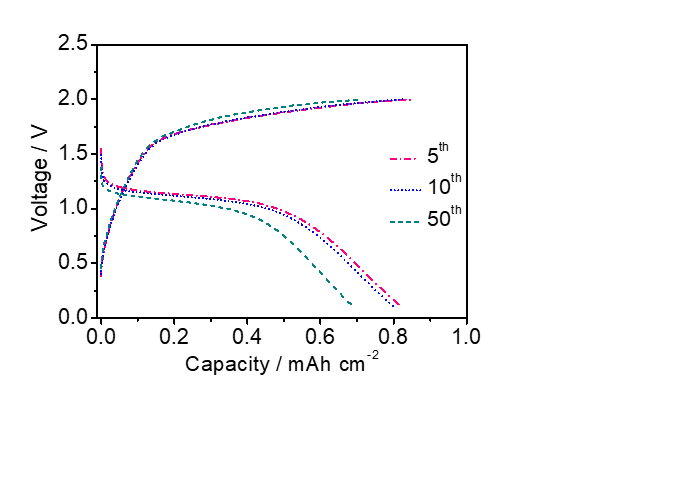


**Supplementary Figure 33**. The voltage profiles of the S/C|gel electrolyte|Ca_0.4_MnO_2_ pouch cell during 50 cycles at 0.2 C. The mass ratio of Ca_0.4_MnO_2_ cathode to S/C anode is about 1.6: 1.


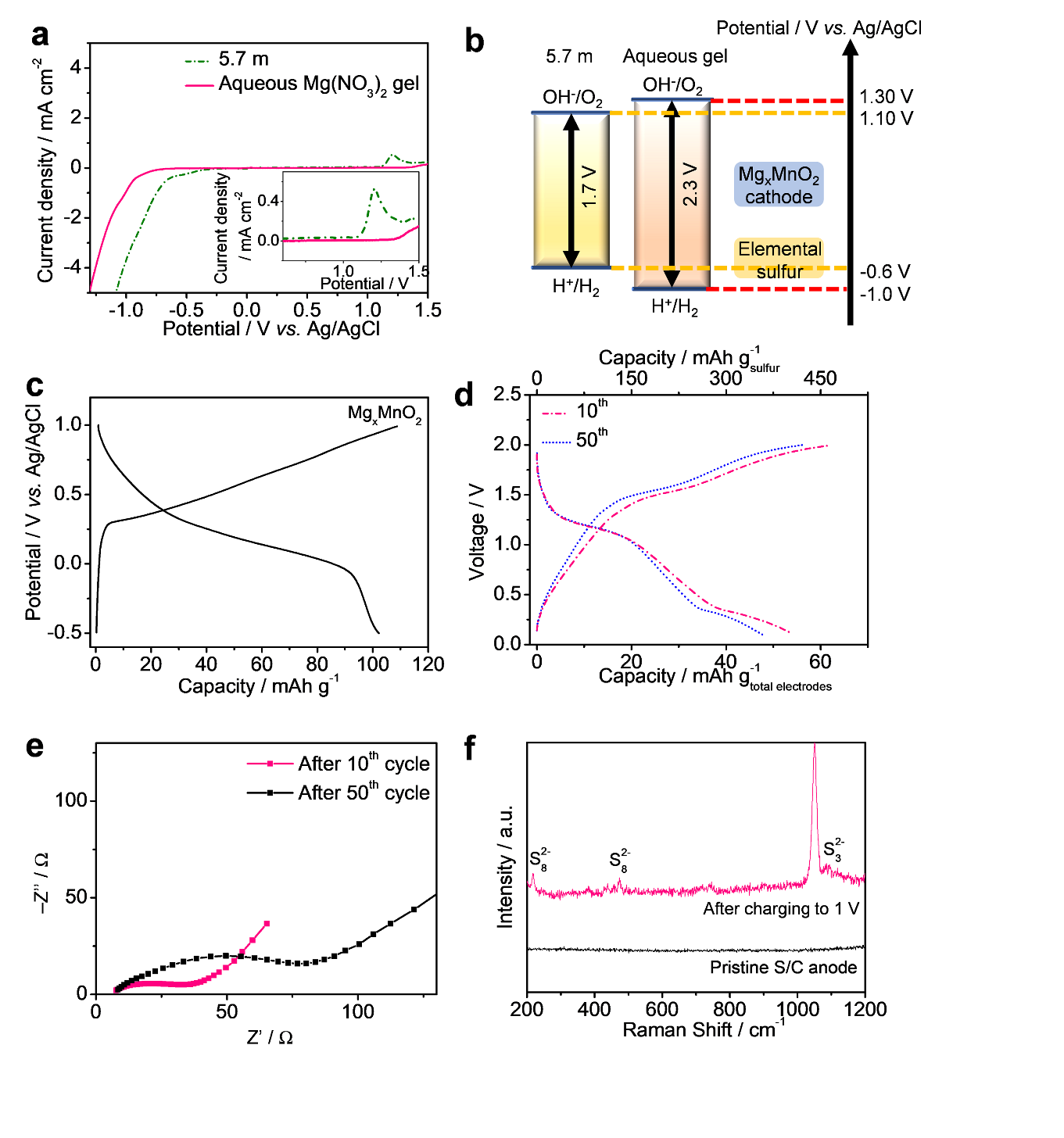


**Supplementary Figure 34**. The performances of the aqueous Mg–ion/sulfur||metal oxide batteries. **a** Linear voltammetry curves recorded at 1 mV s^–1^ in saturated (5.7 m) Mg(NO_3_)_2_ electrolyte, and saturated Mg(NO_3_)_2_ aqueous solution with 10 wt% PVA gel electrolyte (aqueous Mg(NO_3_)_2_ gel electrolyte). A magnified view of the region outlined near the cathodic extreme is shown in inset. **b** The electrochemical stability windows of electrolytes, and the redox voltages of Mg_x_MnO_2_ cathode and sulfur anode. **c** Voltage profiles of the Mg_x_MnO_2_ cathode at a specific current of 100 mA g^–1^. The Mg_x_MnO_2_ cathode was prepared by pre–magnesiating the Mn_3_O_4_ electrodes by charging–discharging the electrochemical cell within a voltage window of –0.5~1.0 V *vs.* Ag/AgCl at 50 mA g^–1^ for 10 cycles in the presence of saturated aqueous Mg(NO_3_)_2_. The Mg_x_MnO_2_ cathode can deliver a reversible capacity of about 100 mAh g^–1^. **d** The voltage profiles of S/C|aqueous Mg(NO_3_)_2_ gel electrolyte|Mg_x_MnO_2_ cell at 0.2 C rate (1 C = 1675 mAh g^–1^ based on the sulfur mass). The aqueous cells were assembled following a same procedure as that of the ACSB with a mass ratio (cathodes to anodes) of about 2: 1. The full cell can deliver a reversible capacity of 360 mAh g^–1^ based on sulfur mass after 50 cycles. **e** The EISs of the S/C|aqueous Mg(NO_3_)_2_ gel electrolyte|Mg_x_MnO_2_ cell after different cycles. The full cell maintains stable electrode|electrolyte interfaces with small resistance during cycling. **f** Raman spectra of pristine S/C anode, and the S/C anode disassembled from the S/C|aqueous Mg(NO_3_)_2_ gel electrolyte|Mg_x_MnO_2_ cell after charging to 1 V. The Raman spectra confirm the conversion chemistry of sulfur anode in the aqueous Mg–ion/sulfur||metal oxide batteries.


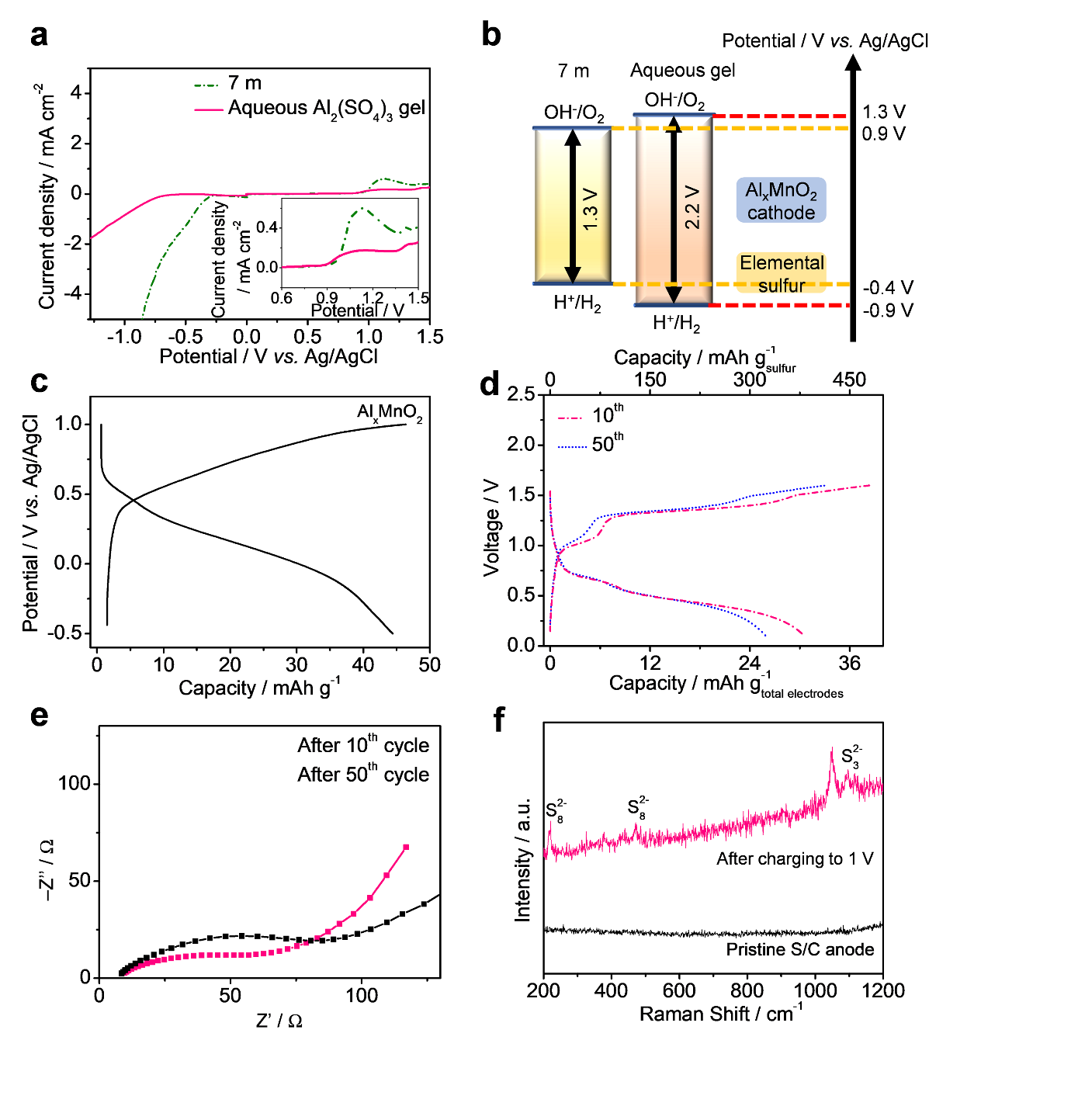


**Supplementary Figure 35**. The performances of the aqueous Al–ion/sulfur||metal oxide batteries. **a** Linear voltammetry curves recorded at 1 mV s^–1^ in saturated (7 m) Al_2_(SO_4_)_3_ electrolyte, and saturated Al_2_(SO_4_)_3_ aqueous solution with 10 wt% PVA gel electrolyte (aqueous Al_2_(SO_4_)_3_ gel electrolyte). A magnified view of the region outlined near the cathodic extreme is shown in inset. **b** The electrochemical stability windows of electrolytes, and the redox voltages of Al_x_MnO_2_ cathode and sulfur anode. **c** Voltage profiles of the Al_x_MnO_2_ cathode at a specific current of 100 mA g^–1^. The Al_x_MnO_2_ cathode was prepared by pre–aluminating the Mn_3_O_4_ electrodes by charging–discharging the electrochemical cell within a voltage window of –0.5~1.0 V *vs.* Ag/AgCl at 50 mA g^–1^ for 10 cycles in the presence of saturated aqueous Al_2_(SO_4_)_3_. The Al_x_MnO_2_ cathode can deliver a reversible capacity of about 45 mAh g^–1^. **d** The voltage profiles of S/C|aqueous Al_2_(SO_4_)_3_ gel electrolyte|Al_x_MnO_2_ cell at 0.2 C rate (1 C = 1675 mAh g^–1^ based on the sulfur mass). The aqueous cells were assembled following a same procedure as that of the ACSB with a mass ratio (cathodes to anodes) of about 4: 1. The full cell can deliver a reversible capacity of 315 mAh g^–1^ based on sulfur mass after 50 cycles. **e** The EISs of the S/C|aqueous Al_2_(SO_4_)_3_ gel electrolyte|Al_x_MnO_2_ cell after different cycles. The full cell maintains stable electrode|electrolyte interfaces with small resistance during cycling. **f** Raman spectra of pristine S/C anode, and the S/C anode disassembled from the S/C|aqueous Al_2_(SO_4_)_3_ gel electrolyte|Al_x_MnO_2_ cell after charging to 1 V. The Raman spectra confirm the conversion chemistry of sulfur anode in the aqueous Al–ion/sulfur||metal oxide batteries.


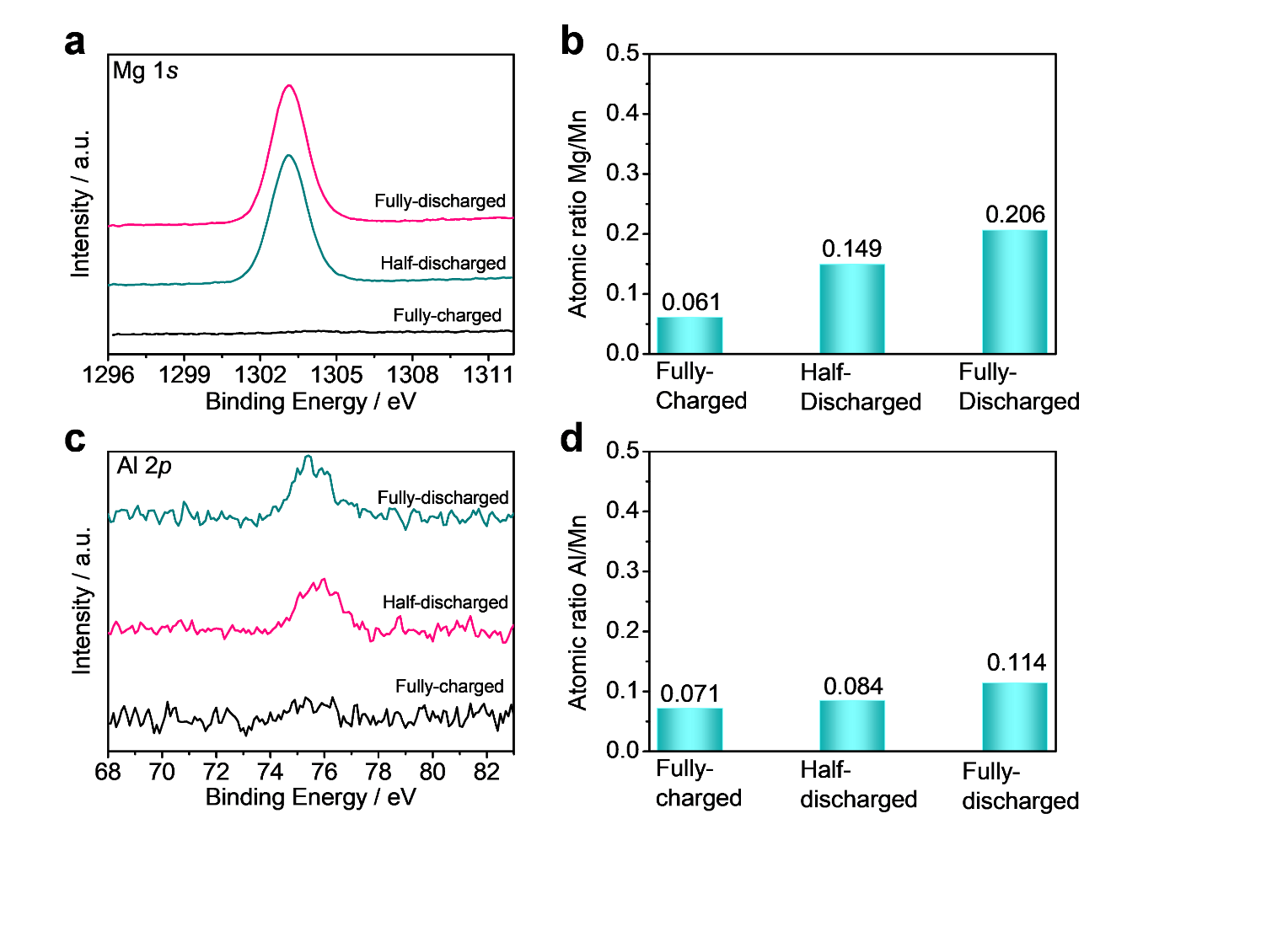


**Supplementary Figure 36. a** The XPS spectra of the Mg_x_MnO_2_ cathode at different states of charge. **b** The atomic ratio of Mg and Mn according to ICP results. **c** The XPS spectra of the Al_x_MnO_2_ cathode at different states of charge. **d** The atomic ratio of Al and Mn according to ICP results. The cathode samples were pre–heated at 80 °C for one day before characterizations.


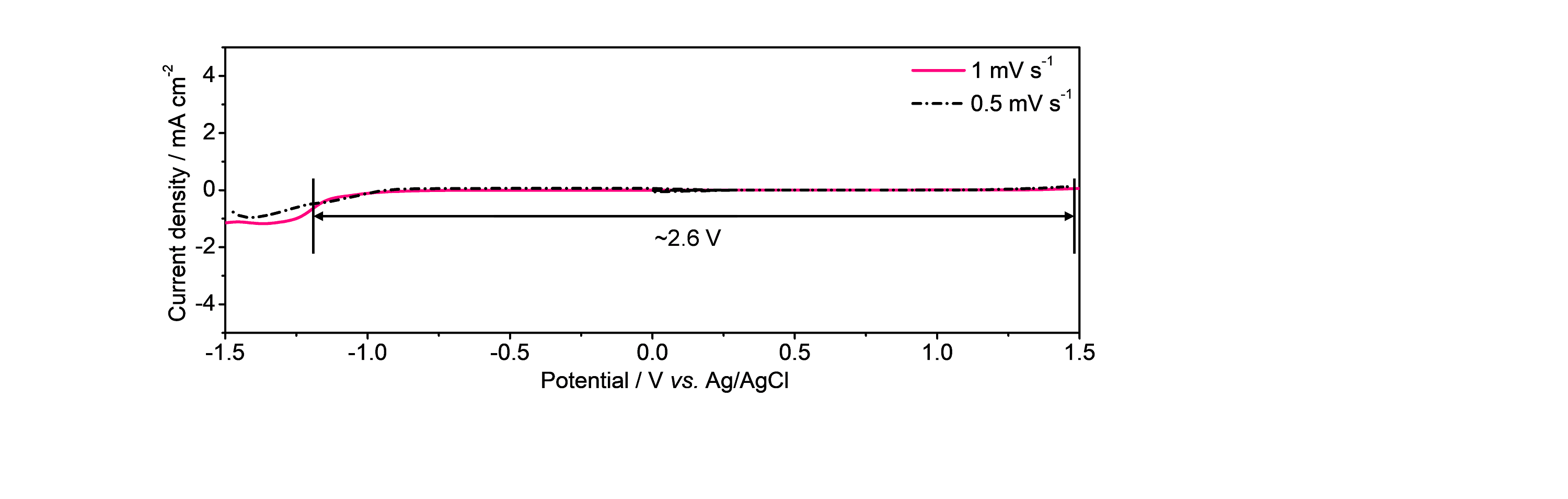


**Supplementary Figure 37**. Linear voltammetry curves of aqueous gel electrolyte recorded at 0.5 mV s^–1^ and 1 mV s^–1^ (See discussion in Supplementary Note 9).

**Supplementary Notes**

**Supplementary Note 1.** Supplementary Fig. 4 shows the radial distribution functions of distance between two oxygen atoms in water molecules in different electrolytes. It can be observed that a sharp peak located at ≈2.75 Å in dilute solution represents the d(O_w_–O_w_) between two nearest water molecules connected by a hydrogen bond, while this peak become broader and weaker in Sat. solution and gel electrolyte, which means the two nearest water molecules may be coordinated with a Ca^2+^ ion to form a Ca^2+^–H_2_O complex.

**Supplementary Note 2.** As shown in Supplementary Fig. 7, the average binding energy between one water molecule and Ca^2+^ is about 0.78 eV, while the average water–water binding energy is only about 0.27 eV^3^. Therefore, the water molecules are preferentially bonded by Ca^2+^, which significantly decrease the free water molecules and increase Ca^2+^–O_H_ (water oxygen) interaction. This result is well consistent with the ^1^H NMR spectra in Supplementary Fig. 6.

**Supplementary Note 3.** As shown in the UV–vis spectra of the 1 m and 8.37 m Ca(NO_3_)_2_ aqueous electrolytes with/without adding S/C electrodes (Supplementary Fig. 8), the pristine electrolytes (*e.g.* 1 m and 8.37 m Ca(NO_3_)_2_ aqueous solutions) present a peak at ≈305 nm, which is assigned to the Ca(NO_3_)_2_ salt^4^. After immersing S/C electrodes, no newly formed peaks were observed in the UV–vis spectra. This result indicates that the solubility of elemental sulfur is negligible in the aqueous electrolytes.

**Supplementary Note 4.** As can be seen from Supplementary Fig. 12, the S 2*p* XPS spectra can be fitted into four peaks at 162.3, 166.3, 168.2, 170.7 eV, which can be attributed to the CaS, SO_3_^2–^, polythionate complex, and HS^–^, respectively^5–7^. Particularly, the polythionate complex and SO_3_^2–^ may result from the interfacial chemistry.

**Supplementary Note 5.** Supplementary Fig. 22a displays the CV curves of the cell at various scan rates from 0.2 to 0.5 mV s^–1^. From these CV curves, we can obtain a plot of log(peak current) as function of log(scan rate) based on equation (S1) and (S2)^8^:

*i* = a*ν*^b^ (S1)

log(*i*) = blog(*ν*) + log(a) (S2)

where *i* is peak current, *ν* is scan rate, a and b are adjustable parameters. The value of b is determined by the slope in the log(*ν*)–log(*i*) plots. The b values in Supplementary Fig. 22b are calculated to be 0.53 for anodic peak, indicating that the capacity at peak voltage is dominated by the non–capacitive behaviour^8,9^.

**Supplementary Note 6.** Galvanostatic intermittent titration technique (GITT) has been applied to examine the Ca^2+^ diffusion in the Ca_0.4_MnO_2_ cathode materials. The obtained GITT curve of Ca_0.4_MnO_2_ cathode is shown in Supplementary Fig. 23b and 25a. The $D_{{Ca}^{2+}}$ can be obtained via the following equation^10,11^:

$D_{{Ca}^{2+}}= \frac{4}{\pi\tau}{(\frac{m_{B}V_{M}}{M_{B}S})}^{2}{(\frac{{\triangle E}_{s}}{{\triangle E}_{\tau}})}^{2}$ (S3)

where the weight of active material (*i. e.* Ca_0.4_MnO_2_), 𝑚_B_, is 10.24 mg, and molar mass (𝑀_B_)/molar volume (𝑉_M_) are calculated to be about 3.4^12^. 𝑆 is the contact area between electrolyte and electrode, which is approximated as the surface area of electrode (0.385 cm^2^). 𝛥𝐸_s_ is IR drop and 𝛥𝐸_τ_ is the potential change in the time range of τ (s) during the discharge process (Supplementary Fig. 25b). As shown in Supplementary Fig. 25c, τ^0.5^ shows a good linear relationship with potential, which is the pre–condition of the equation S3. The Ca^2+^ diffusion co–efficiency is as high as 9.58 $\times$ 10^–8^ at 1 V *vs.* Ag/AgCl, domanstrating a rapid Ca^2+^ diffusion in the layered MnO_2_. This value decreases to 1.23 $\times$ 10^–10^ at –0.29 V, which is asbribed to the intercalation of Ca^2+^ into the layered structure of cathode materials.

**Supplementary Note 7.** EIS is applied to evaluate the interfacial stability of the cathode after different cycles. Supplementary Fig. 26 presents the Nyquist plots of the cathode materials after 2^nd^ cycle and 50^th^ cycle. The Nyquist plots exhibit a semicircle in the high–frequency region and a slope line at the low–frequency region. The slope line with an angle of about 45° suggests the diffusion–control process. The diameter of semicircles reflects the charge–transfer resistance (*R_ct_*) between electrolyte and cathode materials. Additionally, the intercept at real axis represents the bulk resistance (*R_b_*)^13,14^. Only a small increase of *R_ct_* from 20 to 60 Ω is observed after 50 cycles. These EIS results further confirm the good interfacial stability of the cathode electrode.

**Supplementary Note 8.** We employed multiple characterization methods to clarify the cation intercalation/de–intercalation mechanism of Ca_0.4_MnO_2_ cathode in aqueous gel electrolyte. According to the thermogravimetry analysis (TGA) in Supplementary Fig. 27a, the mass loss in the range from 150 ~300 ^o^C is attributed to the evaporation of lattice water molecules, which is confirmed by the mass spectrum result shown in the inset of Supplementary Fig. 27a^15^. During the discharge process, the lattice water content of cathode increases to 3.1 % in the fully–discharged state, indicating the co–insertion of water molecules. This is consistent with the Fourier–transform infrared spectroscopy (FTIR) results (Supplementary Fig. 27b**)**, in which the peak at 3500 cm^–1^ ascribed to the aggregated H_2_O molecules becomes stronger after the discharge process^16^. Additionally, as seen from the ^1^H solid state nuclear magnetic resonance (SSNMR) spectra in Supplementary Fig. 27c, the peak at ≈1.0 ppm can be attributed to the co–intercalated proton in the cathode lattice, meanwhile the peaks at ≈2.5 and 7.2 ppm are assigned to the inserted H_2_O molecules^17,18^. The intensities of both the proton and H_2_O peaks gradually increase during the discharge process, demonstrating the co–intercalation of protons into the cathode lattice.

Furthermore, XPS measurements were also conducted on the Ca_0.4_MnO_2_ cathodes after cycled in the aqueous gel electrolyte. We find that the intensity of the Ca^2+^ peak at 347.4 and 350.8 eV gradually increases during the discharge process of cathode (Supplementary Fig. 27d), and the calculated specific capacity generated from the reversible intercalation of Ca^2+^ in the cathode is ≈ 109 mAh g^–1^ based on the Ca/Mn atomic ratio change from the inductively coupled plasma (ICP) results (Supplementary Fig. 27e). According to above discussions, the electrochemical mechanism of Ca_0.4_MnO_2_ cathode is presented in Supplementary Fig. 27f. During the discharge process, protons and H_2_O molecules first co−insert into the lattice, which contributes a small proportion of the capacity. Particularly, the presence of water molecules in the cathode lattice efficiently decreases the cation diffusion barrier, thus improving the kinetics of batteries^19^. As a result, the Ca^2+^ ions can reversibly intercalate into the cathode during the discharge process with improved kinetics, which contributes a major proportion of the total capacity (≈ 80.7 %). This mechanism has been proved by the recent operando pH measurements for aqueous multivalent ion batteries by S. Passerini *et al.*^20^*.* This confirms that protons and multivalent metal cations co–participate in the co–intercalation process and the reversible insertion/extraction of multivalent metal cations in the manganese oxide cathodes makes a major contribution to the battery capacities.

**Supplementary Note 9.** In this work, the electrochemical stability window was recorded as the voltage range where the HER current density was lower than 0.5 mA cm^–2^ and OER current density was lower than 0.1 mA cm^–2^. The electrochemical stability window of the gel electrolyte is ≈2.6 V at a scan rate of 1 mV s^–1^. When decreasing the scan rate to 0.5 mV s^–1^, the electrochemical stability window still remains ≈2.6 V, which can support the redox reaction of Ca_0.4_MnO_2_ cathode and S/C anode in the full cell (Supplementary Fig. 37). Therefore, the scan rate of LSV was set as 1 mV s^–1^ in this work.

**Supplementary Tables**

**Supplementary Table 1.** ICP results of the cathode material after electrochemical transformation.

|  | Ca | Mn |
| --- | --- | --- |
| Molar ratio | 0.398 | 1 |

**Supplementary Table 2**. The comparison of the electrochemical performance of the aqueous battery systems.

|  | Specific Energy (Wh kg^–1^) | Specific Power (W kg^–1^) | Durability | Ref. |
| --- | --- | --- | --- | --- |
| KFeMnHCF\|\|PTCDI | 80 | 41 | 87% after 500 cycles; 73% after 2000 cycles | 21 |
| NaCuHCF\|\|NTP | 48.3 | 91 | 97% after 100 cycles | 22 |
| CuHCF\|\|MnHCF | 15 | 693 | No Capacity loss after 1000 cycles | 23 |
| NaMnO_2_\|\| NaTi_2_(PO_4_)_3_ | 30 | 50 | 75% after 500 cycles | 24 |
| CaCuHCF\|\|PNDIE | 54 | 48 | 88% after 50 cycles | 25 |
| PB\|\|Polyimide | ~35 | 200 | 60% after 2000 cycles | 26 |
| PPMDA\|\|LVP | 55 | 106 | 86.8% after 1000 cycles | 27 |
| CuHCF\|\|MoO_3_ | 21 | 350 | 63.7% after 100 cycles | 28 |
| S/C\|\|Ca_0.4_MnO_2_ | 110 | 33 | 83% after 150 cycles | This work |

**Supplementary Table 3**. Force field parameters used for Ca(NO_3_)_2_ aqueous solution in molecular dynamics simulations.

| Atom type | Charge (e) | σ (Å) | ε (kcal mol^–1^) |
| --- | --- | --- | --- |
| H | 0.4238 | 0 | 0 |
| O (water) | –0.8476 | 3.166 | 0.1553 |
| Ca | 1.5 | 2.412 | 0.450 |
| O (NO^3–^) | –0.4485 | 2.771 | 0.1456 |
| N | 0.5955 | 3.059 | 0.08066 |

The compositions of simulated electrolytes are given in the following table. The velocity–Verlet algorithm is applied to integrate the equations of motion with a time step equalled to 1 fs. First, NPT runs were performed at 500 K for 2 ns and then 298 K for 7 ns to ensure that the equilibrium salt dissociation had been reached. Then, the NPT runs were controlled by Nose–Hooven thermostat and barostat with a 100 fs damping parameter. Then, the NVT runs were 10 ns long at 298K. At this state, all simulated systems were surely equilibrated. The hydrogen bonds are defined by a distance cutoff of 3.5 Å and an angle cutoff of 30°.

**Supplementary Table 4**. Compositions of simulated electrolytes.

|  | 1 m Ca(NO_3_)_2_ | Sat. Ca(NO_3_)_2_ | Gel electrolyte |
| --- | --- | --- | --- |
| Number of H_2_O per box | 1913 | 1434 | 1429 |
| Number of Ca(NO_3_)_2_ per box | 34 | 216 | 215 |
| Equilibrium volume(Å3) | 59822 | 62254 | 72715 |
| T(K) | 298 | 298 | 298 |
| MD, density(g cm^–3^) | 1.11 | 1.63 | 1.55 |

**Supplementary Methods**

***Computation.*** Molecular dynamics (MD) simulations were performed to investigate the structures of aqueous solutions/gel electrolyte and the diffusion of polysulfides as a function of salt concentration. The MD simulations were run by using LAMMPS^29^. The systems are setup initially by using PACKMOL^30^ and Moltemplate (http://www.moltemplate.org/). Periodic boxes were used here. The properties of H_2_O are assessed with SPC/E parameters. The force–fields parameters of Ca^2+^ and NO_3_^−^ are taken from previous publications^31,32^ with partial charges (shown in Supplementary Table 3). The compositions of simulated electrolytes are given in the Supplementary Table 4. The force–fields parameters of PVA chains and polysulfides are taken from OPLS–AA parameters OPLS–AA parameters and previously publications^33^. Herein, an oligomer (CH_3_[C_2_H_4_O]_4_) form was utilized to simplify the gel electrolyte simulations. A LJ cutoff of 10 Å and a particle–particle particle–mesh solver^34^ for long–range Coulombic interactions were also employed.

Density functional theory (DFT) calculations were employed to study the reduction potentials of Ca^2+^(NO_3_^–^)_3_(H_2_O)_x_ complex and isolated NO_3_^–^ as well as the binding energy. The calculations of reduction potentials and binding energy were performed with Gaussian 16 package^34^. All calculations were carried out with a solvation correction under SMD model^35^. All the thermal dynamic results were obtained with a combined method of G4(MP2)^36^, and the final results were corrected as 298 K. The reduction potentials E(NO_3_^–^/NO_2_^–^) *vs.* the SHE were calculated according to the reported method^37^ from the half reactions:

NO_3_^–^ + 2e^–^ → NO_2_^–^ + O^2–^ ΔG (S4)

and

H^+^ + e^–^ → 1/2H_2_ ΔG_SHE_ (S5)

in which NO_3_^–^ and NO_2_^–^ are the oxidized and reduced iron species in the reduction, and ΔG and ΔG_SHE_ are the aqueous free energy changes for the respective half reactions, ignoring the electron. The final reduction potential (*vs.* SHE) was calculated with the following equation:

$E= -(\frac{\Delta G}{zF}-\frac{\Delta G_{SHE}}{F}$) (S6)

where in the reaction, *z* is the charge transfer and F is Faraday constant. The binding energies were calculated using wB97xd functionals^38^ with the basis sets of def2–TZVPP^39^.

**Supplementary References**

1. Manan, N. S. *et al.* Electrochemistry of sulfur and polysulfides in ionic liquids. *J. Phys. Chem. B* **115**, 13873–13879 (2011).
2. Yang, C. P., Yin, Y. X., Guo, Y. G. & Wan, L. J. Electrochemical (de) lithiation of 1D sulfur chains in Li–S batteries: a model system study. *J. Am. Chem. Soc.* **137**, 2215–2218 (2015).
3. Zheng, J. *et al.* Understanding thermodynamic and kinetic contributions in expanding the stability window of aqueous electrolytes. *Chem* **4**, 2872–2882 (2018).
4. Hudson, P. K., Schwarz, J., Baltrusaitis, J., Gibson, E. R. & Grassian, V. H. A spectroscopic study of atmospherically relevant concentrated aqueous nitrate solutions. *J. Phys. Chem. A* **111**, 544–548 (2007).

5 Liang, X. *et al.* A highly efficient polysulfide mediator for lithium–sulfur batteries. *Nat. Commun.* **6**, 1–8 (2015).

6 Franzen, H. F., Umaña, M. X., McCreary, J. & Thorn, R. XPS spectra of some transition metal and alkaline earth monochalcogenides. *J. Solid State Chem.* **18**, 363–368 (1976).

7 Cavell, R. G. & Sodhi, R. N. Absolute total static relaxation and extra–atomic relaxation terms for volatile phosphorus compounds obtained from the Auger parameter and relativistic. *J. Electron. Spectros. Relat. Phenomena* **41**, 25–35 (1986).

8 Brezesinski, T., Wang, J., Tolbert, S. H. & Dunn, B. Ordered mesoporous α–MoO_3_ with iso–oriented nanocrystalline walls for thin–film pseudocapacitors. *Nat. Mater.* **9**, 146–151 (2010).

9 Wang, J., Polleux, J., Lim, J. & Dunn, B. Pseudocapacitive contributions to electrochemical energy storage in TiO_2_ (anatase) nanoparticles. *J. Phys. Chem. C* **111**, 14925–14931 (2007).

10 Zhu, Y. & Wang, C. Galvanostatic intermittent titration technique for phase–transformation electrodes. *J. Phys. Chem. C* **114**, 2830–2841 (2010).

11 Dees, D. W., Kawauchi, S., Abraham, D. P. & Prakash, J. Analysis of the galvanostatic intermittent titration technique (GITT) as applied to a lithium–ion porous electrode. *J. Power Sources* **189**, 263–268 (2009).

12 Bach, S., Pereira–Ramos, J. & Baffier, N. A new MnO_2_ tunnel related phase as host lattice for Li intercalation. *Solid State Ionics* **80**, 151–158 (1995).

13 Zhang, X. *et al.* Rapid hydrothermal synthesis of hierarchical nanostructures assembled from ultrathin birnessite–type MnO_2_ nanosheets for supercapacitor applications. *Electrochim. Acta* **89**, 523–529 (2013).

14 Gao, H., Xiao, F., Ching, C. B. & Duan, H. High–performance asymmetric supercapacitor based on graphene hydrogel and nanostructured MnO_2_. *ACS Appl. Mater. Interfaces* **4**, 2801–2810 (2012).

15 Meija, J., Mester, Z. & D’Ulivo, A. Mass spectrometric separation and quantitation of overlapping isotopologues. H_2_O/HOD/D_2_O and H_2_Se/HDSe/D_2_Se mixtures. *J. Am. Soc. Mass Spectrom.* **17**, 1028–1036 (2006).

16 Scatena, L., Brown, M. & Richmond, G. Water at hydrophobic surfaces: Weak hydrogen bonding and strong orientation effects. *Science* **292**, 908–912 (2001).

17 Sa, N. *et al.* Is alpha–V_2_O_5_ a cathode material for Mg insertion batteries? *J. Power Sources* **323**, 44–50 (2016).

18 Fărcaşiu, D., Lukinskas, P. & Hâncu, D. The hydronium tetrafluoroborate dimer in nonpolar media and its proton NMR spectrum. *J. Mol. Model.* **6**, 171–176 (2000).

19 Wang, F. *et al.* How water accelerates bivalent ion diffusion at the electrolyte/electrode interface. *Angew. Chem. Int. Ed.* **57**, 11978–11981 (2018).

20 Liu, X. *et al.* Operando pH measurements decipher H^+^/Zn^2+^ intercalation chemistry in high–performance aqueous Zn/δ–V_2_O_5_ batteries. *ACS Energy Lett.* **5**, 2979–2986 (2020).

21 Jiang, L. *et al.* Building aqueous K–ion batteries for energy storage. *Nat. Energy* **4**, 495–503 (2019).

22 Wu, X. y. *et al.* Energetic Aqueous Rechargeable Sodium‐Ion Battery Based on Na_2_CuFe(CN)_6_–NaTi_2_(PO_4_)_3_ Intercalation Chemistry. *ChemSusChem* **7**, 407–411 (2014).

23 Pasta, M. *et al.* Full open–framework batteries for stationary energy storage. *Nat. Commun.* **5**, 1–9 (2014).

24 Hou, Z., Li, X., Liang, J., Zhu, Y. & Qian, Y. An aqueous rechargeable sodium ion battery based on a NaMnO_2_–NaTi_2_(PO_4_)_3_ hybrid system for stationary energy storage. *J. Mater. Chem. A* **3**, 1400–1404 (2015).

25 Gheytani, S. *et al.* An aqueous Ca‐ion battery. *Adv. Sci.* **4**, 1700465 (2017).

26 Chen, L. *et al.* Aqueous Mg–ion battery based on polyimide anode and Prussian blue cathode. *ACS Energy Lett.* **2**, 1115–1121 (2017).

27 Wang, F. *et al.* High–voltage aqueous magnesium ion batteries. *ACS Cent. Sci.* **3**, 1121–1128 (2017).

28 Wang, P. *et al.* A flexible aqueous Al ion rechargeable full battery. *Chem. Eng. J.* **373**, 580–586 (2019).

29 Plimpton, S. Computational limits of classical molecular dynamics simulations. *J. Comput. Phys.* **117**, 361–364 (1995).

30 Martínez, L., Andrade, R., Birgin, E. G. & Martínez, J. M. PACKMOL: a package for building initial configurations for molecular dynamics simulations. *J. Comp. Chem.* **30**, 2157–2164 (2009).

31 Zheng, J. *et al.* Understanding thermodynamic and kinetic contributions in expanding the stability window of aqueous electrolytes. *Chem* **4**, 2872–2882 (2018).

32 Kohagen, M., Mason, P. E. & Jungwirth, P. Accurate description of calcium solvation in concentrated aqueous solutions. *J. Phys. Chem. B* **118**, 7902–7909 (2014).

33 Jorgensen, W. L., Maxwell, D. S. & Tirado–Rives, J. Development and testing of the OPLS all–atom force field on conformational energetics and properties of organic liquids. *J. Am. Chem. Soc.* **118**, 11225–11236 (1996).

34 Hockney, R. W. & Eastwood, J. W. *Computer simulation using particles*. (crc Press, 1988).

35 Marenich, A. V., Cramer, C. J. & Truhlar, D. G. Universal solvation model based on solute electron density and on a continuum model of the solvent defined by the bulk dielectric constant and atomic surface tensions. *J. Phys. Chem. B* **113**, 6378–6396 (2009).

36 Curtiss, L. A., Redfern, P. C. & Raghavachari, K. Gaussian–4 theory. *J. Chem. Phys.* **126**, 084108 (2007).

37 Ali–Torres, J., Rodriguez–Santiago, L., Sodupe, M. & Rauk, A. Structures and stabilities of Fe^2+^/^3+^ complexes relevant to Alzheimer’s disease: an ab initio study. *J. Phys. Chem. A* **115**, 12523–12530 (2011).

38 Chai, J.–D. & Head–Gordon, M. Long–range corrected hybrid density functionals with damped atom–atom dispersion corrections. *Phys. Chem. Chem. Phys.* **10**, 6615–6620 (2008).

39 Weigend, F., Furche, F. & Ahlrichs, R. Gaussian basis sets of quadruple zeta valence quality for atoms H–Kr. *J. Chem. Phys.* **119**, 12753–12762 (2003).
