# Supplementary material for: A universal strategy towards high–energy aqueous multivalent–ion batteries
Source: Nat Commun. 2021 May 17;12:2857. doi: 10.1038/s41467-021-23209-6 (PMC8128864; doi:10.1038/s41467-021-23209-6)
Supplement: Supplementary file 3 — Description of Additional Supplementary Files [file 41467_2021_23209_MOESM3_ESM.docx]

Description of additional supplementary information files

Title: Supplementary Movie 1

Description: Diffusion of a typical soluble polysulfide (CaS4) in 1 m Ca(NO3)2 aqueous electrolyte.

Title: Supplementary Movie 2

Description: Diffusion of a typical soluble polysulfide (CaS4) in 8.37 m Ca(NO3)2 aqueous electrolyte.

Title: Supplementary Movie 3

Description: Diffusion of a typical soluble polysulfide (CaS4) in aqueous gel electrolyte.

Title: Supplementary Movie 4

Description: Water–soaking test of charged S/C|aqueous gel electrolyte|Ca0.4MnO2 pouch cell after corner–cut.
